# Detection and Quantification of Histone H4 Citrullination in Early NETosis With Image Flow Cytometry Version 4

**DOI:** 10.3389/fimmu.2020.01335

**Published:** 2020-07-16

**Authors:** Emilia A. Barbu, Venina M. Dominical, Laurel Mendelsohn, Swee Lay Thein

**Affiliations:** ^1^Sickle Cell Branch, National Heart, Lung, and Blood Institute, NIH, Bethesda, MD, United States; ^2^Flow Cytometry Core Facility, National Heart, Lung, and Blood Institute, NIH, Bethesda, MD, United States

**Keywords:** PMN, NETs, H4cit3, imaging flow cytometry, stimulus-dependent

## Abstract

Neutrophil extracellular traps (NETs) formation has been implicated in an increasing number of infectious and non-infectious pathologies. NETosis is a tightly regulated process; the end-stage and read-out is the formation of DNA strands extruded from the nuclei, and traditionally assessed by fluorescence microscopy. Since NETosis has emerged as a possible biomarker of the inflammatory process, there is a need for less time-consuming, consistent, and quantitative approaches to improve its application in clinical assessment of pro-inflammatory conditions. Imaging Flow Cytometry (IFC) combines features of conventional flow cytometry with qualitative power of fluorescence microscopy and has an added advantage of the capability of assessing the early processes leading up to extrusion of the DNA-scaffolded strands. We explored the optimal imaging-based tools that can be used to measure citrullination of H4 in early NETosis. IFC identified and quantified histone 4 citrullination (H4cit3) induced with several known NETosis stimuli (Ionophore, PMA, LPS, Hemin, and IL-8) following treatment periods ranging from 2 to 60 min. Its relationship with other alterations at nuclear and cellular level, such as nuclear decondensation and super-condensation, multi-lobulated nuclei vs. 1-lobe nuclei and cell membrane damage, were also quantified. We show that the early progress of the H4cit3 response in NETosis depends on the stimulus. Our method identifies fast (Ionophore and Hemin), intermediate and slow (PMA) inducers and shows that H4cit3 appears to have a limited contribution to both early LPS- and IL-8-induced NETosis. While this method is rapid and of a higher throughput compared to fluorescence microscopy, detection and quantification is limited to H4cit3-mediated nuclear events and is likely to be stimulus- and signaling pathway dependent.

## Introduction

Neutrophils are key players in the innate immunity system. Resting neutrophils patrol the bloodstream and are rapidly recruited to sites of infection and injury upon activation, contributing to host defense, and inflammation. Activated neutrophils respond directly by engulfing the offending microorganism (phagocytosis), by releasing the content of their granules into the environment (degranulation), by producing and releasing reactive oxygen species, or by making extracellular traps (NETs). NETs formation involves chromatin de-condensation and nuclear swelling, transfer of cytoplasmic granules to the nucleus, and mixing of these granules with nuclear material. Eventually, the cell lyses and releases the nuclear/cytoplasmic mix into the environment. The extracellular DNA string-like structures associate with cytoplasmic azurophilic granule components such as elastase (NE) or myeloperoxidase (MPO), two enzymes with significant anti-pathogen properties. NE is a potent serine-protease that can break down the outer membrane of bacteria, and MPO catalyzes production of hypochlorous acid (HOCl), thus contributing to a more effective local antimicrobial activity. While NETs formation is thought to play a critical role in both infectious and sterile inflammation, their contribution to these pathologies, is yet to be determined (1).

NETs are most commonly studied by fluorescence microscopy through identification of specific markers: citrullination of histone 3 or histone 4 and/or extracellular DNA co-localized with NE/MPO. New methodologies are being developed for both research and clinical purposes including ELISA-based assays (2), automated high throughput live detection of nuclear changes (3), and microfluidics (4). Conventional flow cytometry methods are also available although some limitation have been recognized, such as the choice of the probes or how samples are prepared, that can affect gating (5–7). Imaging Flow Cytometry (IFC) allows high-throughput analysis and surpasses the qualitative limitations of conventional flow cytometry. IFC allows visualization of whole cells and combines the analytical power of fluorescent microscopy with the cell detection and statistical robustness of conventional flow cytometry. IFC-based techniques that use nuclear morphological changes to assess NETosis have been described (8–10).

Given the increasingly recognized role of NETosis in infectious and sterile inflammatory environments, it would be pertinent to capture the early processes that leads to the final stage of NETs formation, as this may be relevant with regard to the infectious and non-infectious triggers. We aimed at developing a detection and quantification method that can measure NETs-related markers in minimally processed neutrophils that come from an environment rich in physiological NETs inducers (e.g., activating cytokines, hemolysis, or bacterial-related products). Here, we present an IFC-based method that permits specific detection and quantification of a recognized NETosis marker, histone H4 citrullination (H4cit3) in addition to morphological nuclear changes. Analysis of other parameters, such as nuclear super-condensation, presence of unsegmented nuclei, and cell membrane damage, can be further used to pinpoint toward additional NETs-related characteristics. These analytical parameters were established not only by using a pharmacological inducer (PMA), but also LPS and IL-8 (inducers associated with infectious pathogens) and Hemin (inducer associated with hemolysis). This method might offer a viable alternative of evaluating NETs for both research and clinical studies with an objective of determining whether *in vivo* inhibition of NETs might work as a therapeutic strategy for diverse pathologies.

## Materials, Equipment, and Method

### Human Samples

Samples were obtained from healthy volunteers (study protocol NCT0004799) after obtaining written informed consent; the study was approved by the NHLBI Institutional Review Board.

### Neutrophils Isolation, NETosis Induction, and Quantification

Whole blood was collected in EDTA vacutainers and neutrophils were separated with sterile Polymorphprep gradient medium (Cosmo Bio USA, cat # AXS-1114683) as recommended by the manufacturer. The remaining red blood cells (RBCs) were removed by treatment with ice-cold sterile water for 30 s followed by the addition of sterile KCl 0.6 M. The purified neutrophils were allowed to rest in the incubator, at 37°C, for 30 min.

**Figure 1:**
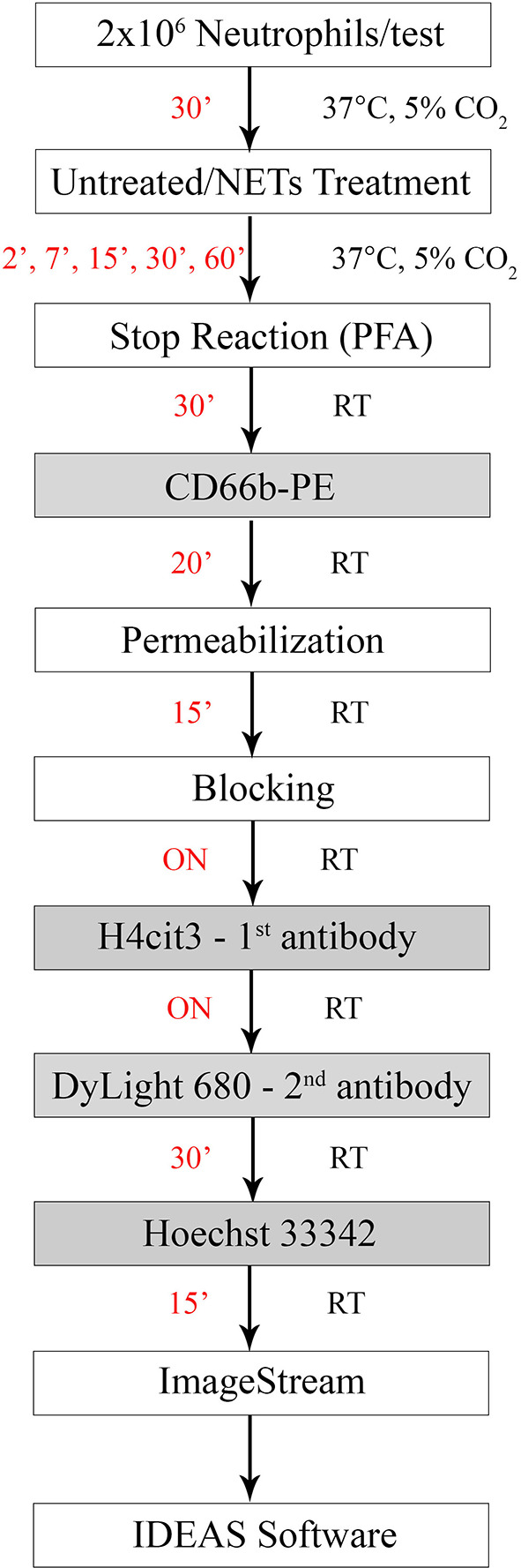
Experimental staining scheme for specific early NETosis detection by imaging flow cytometry. Briefly, purified neutrophils were allowed to rest in the incubator for 30 min (30') prior to the treatment step. NETs agonists were added at the concentrations specified in Table 1 for various times between 2 min (2') and 60 min (60'). Stimulation was stopped by adding paraformaldehyde (PFA) for 30 min (30') at room temperature. Staining steps are underlined in the gray boxes and staining times for each step are in red. Total protocol time from neutrophil isolation to stained samples ready for imaging flow cytometer acquisition is 3 days. (ON, over night; RT, room temperature).

NETosis was induced with several reagents, as described in Table 1. The experimental scheme outlined in Figure 1 describes treatment times with the agonists (between 2 and 60 min), and details of the fixing, permeabilization, and staining times, in minutes at room temperature (RT) or overnight (ON) at 4°C. Donors' contribution to experiments and the number of independent repeats are detailed in Table S1. To assess early histone citrullination response in the absence of an extracellular source of calcium, purified healthy neutrophils were resuspended in DPBS without CaCl_2_ and MgCl_2_, that was supplemented back with 4.5 mM MgCl_2_ and then stimulated with A23187 4 μM as described in Figure 1. (Source of reagents—Calcium Ionophore A23187, Hemin, cat#: 4039 and PMA, cat#: P1585 were from Sigma Aldrich. LPS was from Invitrogen, cat#: tlrl-3pelps and human IL-8 was from Peprotech, cat#: 200-08). Reactions were terminated with 4% PFA and fixed cells were then sequentially stained for CD66b, H4cit3, MPO, and DNA. Briefly, neutrophils were first stained with anti-Human CD66b-PE, clone G10F5 (Biolegend, cat#: 305106) then permeabilized with BD Cytofix/Cytoperm (BD Biosciences, cat#: 554722). A blocking step with 3% BSA in 1xDPBS, no calcium, no magnesium, + 0.2% porcine skin gelatin type A (Sigma, cat#: G1890) was conducted before addition of the primary antibody, rabbit polyclonal anti-histone H4, citrulline 3 (H4cit3, EMD Millipore, cat#: 07-596). Secondary antibody goat anti-Rabbit IgG, DyLight680 (Thermo Fisher, cat#: 35568) and the MPO staining (MPO Polyclonal AlexaFluor 594 Conjugated, Bioss Antibodies, cat#: bs-4943R-A594) were conducted together. The nuclear staining with Hoechst 33342 (BD Pharmingen, cat#: 561908) was last.

**Table 1:** NETs stimuli.

**Reagents**	**Purified neutrophils (2 × 10**^****6****^ **in 500 μl)**						
	**RPMI only (control)**	**Hemin 20 μM**	**Hemin 5 μM**	**LPS 1 μg/ml**	**PMA 100 nM**	**PMA 20 nM**	**IL-8 50 pg/ml**
(μl)	100	100	100	100	100	100	100
Reaction vol. (μl)	600	600	600	600	600	600	600
PFA 8% (μl)	600	600	600	600	600	600	600

For each test 20,000 events were acquired with an ImageStream Mark II imaging flow cytometer (Millipore Sigma, Seattle, WA, USA). Single color controls for each marker were used as compensation controls and unstained cells were used to determine background. While initial tests were conducted including isotype controls, for experimental work FMOs (Fluorescence Minus One) were used to establish proper gating and identify possible unspecific binding or flaws in our panels. Efficiency of the blocking step was confirmed by quantifying the signal for the secondary antibody (DyLight680), in the absence of the primary antibody (H4cit3). Nuclear or cellular changes were identified and quantified by using the IDEAS software. These parameters included histone H4 citrullination signal, MPO colocalization with the nuclear compartment, nuclear decondensation, and super-condensation, and damage of the cellular membrane. Step-by-step acquisition and analysis guide are available in the Supplemental Material.

### Quantification of Apoptotic Neutrophils

Purified neutrophils were treated with Hemin, LPS, PMA, and IL-8 as described in Table 1 and then apoptotic and necrotic cells were detected with a FITC Annexin V Apoptosis Detection Kit with 7-AAD (Biolegend, cat#: 640922). Stained cells were acquired with a BD LSRFortessa (BD Bioscience) and data was analyzed with FlowJo.

### Statistical Analysis

Data is presented as dot plots (RPMI and stimulus) with mean shown or, as percent change from the RPMI sample (i.e., untreated control) with the average value of all repeats per stimulus ± S.D. presented (100 = no change from the untreated control). Statistical analyses were performed with PRISM7 software (Graph Pad Software, CA). Students *t*-test was used for two group comparisons. ANOVA with Bonferroni or Dunnett's post tests were used to compare more than two groups. ^*^P < 0.05, ^**^P < 0.01, ^***^P < 0.001.

## Results

### Progress of H4cit3-Dependent Early NETosis Is Stimulus-Dependent

**Figure 2:**
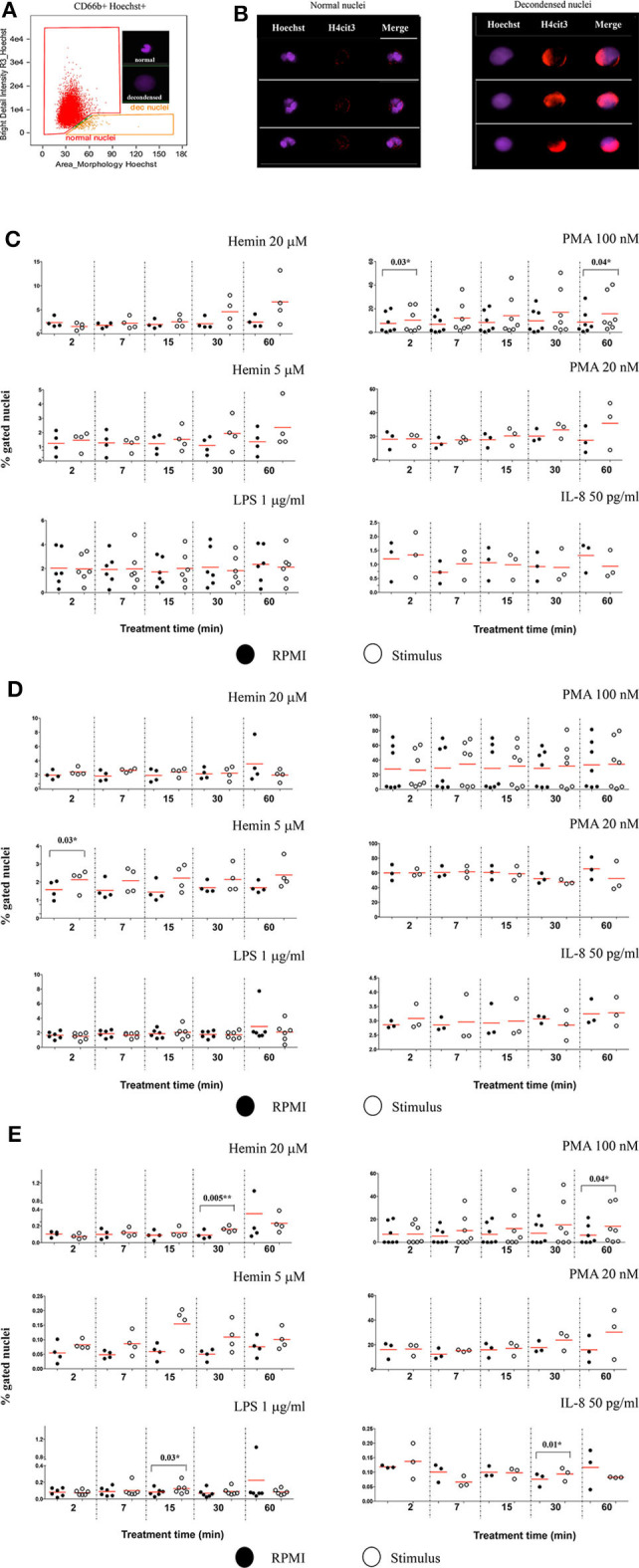
Markers of NETosis can be identified after short and very short periods of treatment with the stimuli. **(A)** Identification of nuclear decondensation using dot-plot of area of the nuclear morphology mask vs. Bright Detail Intensity_R3 feature (normal, multi-lobed nuclei in the red gate, decondensed nuclei in the tangerine gate). **(B)** Examples of neutrophils specimens positive for H4cit3 (red) in the normal nuclei and decondensed nuclei (purple). Purified healthy neutrophils were left untreated in RPMI or treated with the specified NETs stimuli for the indicated lengths of time. **(C)** Percent neutrophils with decondended nuclei. **(D)** H4cit3 positive normal nuclei. **(E)** H4cit3 positive decondensed nuclei. Untreated cells (filled symbols); treated cells (empty symbols). A variable number of experiments were conducted for each stimulus (*N* = 4 for Hemin 20 μM; *N* = 4 for Hemin 5 μM; *N* = 6 for LPS 1 μg/ml; *N* = 7 for PMA 100 nM; *N* = 3 for PMA 20 nM; *N* = 3 for IL-8 50 pg/ml). Paired *t*-test was used to compare the response in untreated and treated neutrophils. *P < 0.05, **P < 0.01.

Changes in nuclear decondensation and histone H4 citrullination (H4cit3)-expressing neutrophils were assessed with imaging flow cytometry (IFC) within 60 min (60′) of treatment with the stimuli. A change in the nuclear morphology from multi-lobular and well-organized to swelled and fuzzy-looking has been regarded as an early marker of neutrophils undergoing NETosis (10). This change can be monitored by using “Bright Detail Intensity_R3” (BDI_R3) feature in the IDEAS software that identifies normal, lobular nuclei as high intensity dye spots with a radius of three pixels or less, and fuzzy, decondensed nuclei as wide areas of low intensity staining (Figure 2A). Examples of H4cit3 positive staining in normal lobulated nuclei, and decondensed nuclei are shown in Figure 2B.

These features have been previously used to detect LPS-induced NETosis (10). We extended it to several stimuli (Hemin, PMA, and IL-8), some at two different concentrations (Hemin at 20 μM and 5 μM and PMA at 100 and 20 nM). We detected LPS- and IL-8-induced minimal changes in nuclear decondensation at any of the five tested time points. In contrast, PMA 100 nM already caused a significant increase in nuclear decondensation after only 2 min (2') of treatment (*P* = 0.03^*^) and another strong increase at 60 min (*P* = 0.04^*^). The two Hemin concentrations needed at least 30 min (30') to induce nuclear decondensation and while the response was clearly increased, the extent was below that elicited by the high PMA concentration and not yet statistically significant (Figure 2C).

We next quantified H4 citrullination (H4cit3), an accepted marker of NETosis, in both normal, segmented nuclei, and in the decondensed ones. With the exception of the two Hemin concentrations, none of the other stimuli induced significant changes in H4cit3 in the normal nuclei. The lower Hemin concentration, 5 μM, caused a rapid and statistically significant increase, only after 2 min of treatment (*P* = 0.03^*^) (Figure 2D). In the decondensed nuclei, IL-8 did not appear to have an effect on H4cit3, while LPS had a limited one (*P* = 0.03^*^ at 15 min), in agreement with the noted lack of nuclear decondensation. PMA 100 nM induced an increase in H4cit3 in a time-dependent manner, significant at the longest treatment time, 60 min (*P* = 0.04^*^). The H4cit3 responses caused by the two Hemin concentrations were clearly increased and statistically significant at longer length of treatment (*P* = 0.005^**^ at 30 min for Hemin 20 μM) (Figure 2E, Figure S1C) and matched an apparent increase in nuclear decondensation at these longer time points. A summary of by-stimulus changes in the nuclear decondensation (Figure S1A) and in the amount of the decondensed nuclei positive for H4cit3 (Figure S1B) confirmed that, following NETs stimulation, more neutrophils responded by increasing H4 citrullination rather than by increasing nuclear decondensation. Generally, the increase in the amount of cells with decondensed and H4cit3+ nuclei was significant at 30 min of treatment. Hemin 20 μM (*P* = 0.02^*^) was more efficient than Hemin 5 μM, while both LPS and IL-8 caused a mild response (*P* = 0.04^*^) (Figure S1B). The lack of nuclear decondensation and H4 citrullination with the LPS and IL-8 stimulations prompted additional testing to confirm strand-like NETs formation. Immunofluorescence microscopy confirmed that NETs agonists that did not elicit responses in the IFC testing (LPS and IL-8 in Figure S2), as well-agonists that caused responses (both Hemin concentrations in Figure S3) did form DNA-Elastase strands. Our data show that while NETs strands are produced following LPS and IL-8 stimulation, this process likely does involve extensive histone 4 citrullination. While our detection method does not provide mechanistic insights, the variability of the readouts suggests that early NETs H4cit3-mediated progression depends on the employed stimulus, and likely on their associated signaling pathways. Follow up studies on the intracellular signaling mediated by TLR-4 (for LPS and Hemin), CXCR1/2 (for IL-8), and PKC (for PMA) should provide additional information on the involvement of these pathways to early NETosis events.

### Calcium Ionophore Is a Fast Inducer of Histone H4 Citrullination in the Absence of Extracellular Calcium

**Figure 3:**
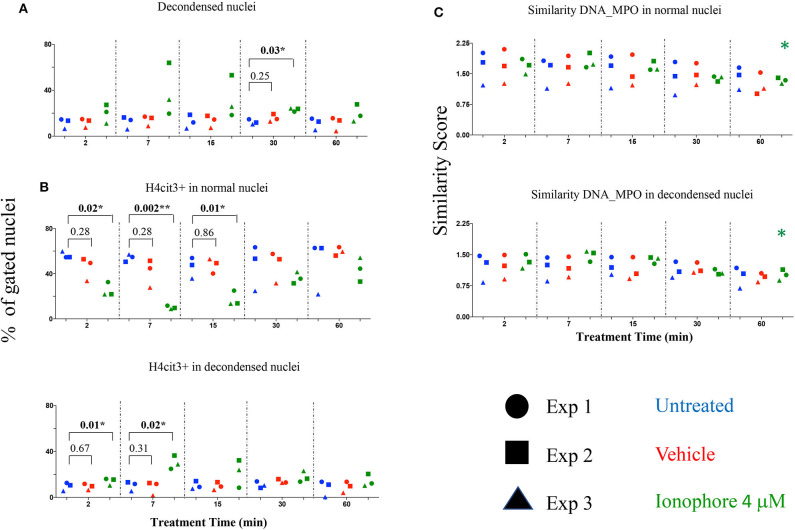
Calcium ionophore treatment induces a rapid change in histone citrullination in both normal and decondensed neutrophil nuclei in the absence of extracellular calcium. Neutrophils purified from three healthy donors were left in DPBS (blue), or treated for the indicated times with vehicle only, DMSO (red), or A23187 4 μM (green) **(A)** Percent neutrophils with decondensed nuclei. **(B)** Percent neutrophils with normal and decondensed nuclei positive for H4cit3. **(C)** Similarity scores for DNA (Hoechst stain) and MPO, marking co-localization of two distinct cellular compartments, nucleus, and type I granules. Paired *t*-test was used to compare untreated with vehicle and ionophore 4 μM, respectively at each time point; Anova for panel ionophore-mediated response in **(C)** *P < 0.05, **P < 0.01.

Calcium ionophore is a noted NETs inducer, with fast kinetics and specific signaling pathways and a requirement for extracellular calcium to form NETs following H3 citrullination (11). As our detection method targets H4 citrullination as a NETs marker we asked whether the presence or absence of an extracellular source of calcium was relevant. Calcium ionophore A23187 was used to stimulate neutrophils without a source of extracellular calcium and nuclear decondensation and H4cit3 in normal and decondensed nuclei were evaluated. We found a significant increase in percent of neutrophils with decondensed nuclei following 30 min of treatment (*P* = 0.03^*^) (Figure 3A). A significant decrease in neutrophils with H4cit3+ normal nuclei was notable after only 2 min of treatment (*P* = 0.02^*^), and remained so for the immediately following tested time points (*P* = 0.002^**^ at 7 min and *P* = 0.01^*^ at 15 min). This decrease was complemented at these short treatment periods by a significant increase in neutrophils with H4cit3+ decondensed nuclei (*P* = 0.01^*^ at 2 min and *P* = 0.02^*^ at 7 min) (Figure 3B). An evaluation of the Mean Fluorescence Intensity (MFI) for H4cit3 in normal nuclei found a significant decrease at 7 min (*P* = 0.04^*^) (Figure S4A). This matched the decrease in H4cit3-expressing cells at this time point. In decondensed nuclei H4cit3 signal intensity decreased significantly after 60 min of treatment (*P* = 0.01^*^) (Figure S4A). By using IFC we were able to confirm the fast kinetics of calcium ionophore-induced NETosis and also show that nuclear changes associated with NETs in the absence of extracellular calcium are H4cit3-dependent.

The recruitment of the primary granules (MPO, elastase) to the nuclear compartment have been associated with NETs formation (12). We explored whether A23187 induced MPO translocation to the nucleus, and how it related with histone H4 citrullination and nuclear decondensation. For this, we employed an IDEAS analysis feature called Similarity, that calculates the degree to which two images (the nuclear stain and the MPO stain) correlated within a target area (i.e., the nuclear area), commonly defined as a mask. Briefly, when MPO is located in the cytoplasm the intensity of its staining is low at the nuclear location, (i.e., it has a dissimilar distribution compared to the Hoechst-stained nuclear mask). When the intensity of both dyes at the nuclear location is high, the image pair has a high degree of similarity and thus, high scores indicate co-localization. We did not confirm higher scores indicating DNA-MPO co-localization after A23187 stimulation, neither in the normal nuclei gate nor in the decondensed nuclei gate (Figure 3C). We did note, that in both normal and decondensed nuclei, the highest scores were found at the shortest stimulation times followed by consistent and time-dependent significant decrease. We asked whether this dissimilarity can be associated with a low amount of MPO available and determined the intensity of its fluorescence signal (Figure S4B). MPO signal was decreased in the cells with normal nuclei after only 2 min of treatment (*P* = 0.03^*^) and remained significantly decreased at 60 min (*P* = 0.01^*^), confirming lower MPO amounts in the cells. As A23187 induces neutrophil degranulation (13), release of the type I granules, containing MPO, might account for the decrease in the MPO availability.

### Nuclear Citrullination but Not MPO Co-Localization Marks Early NETosis

**Figure 4:**
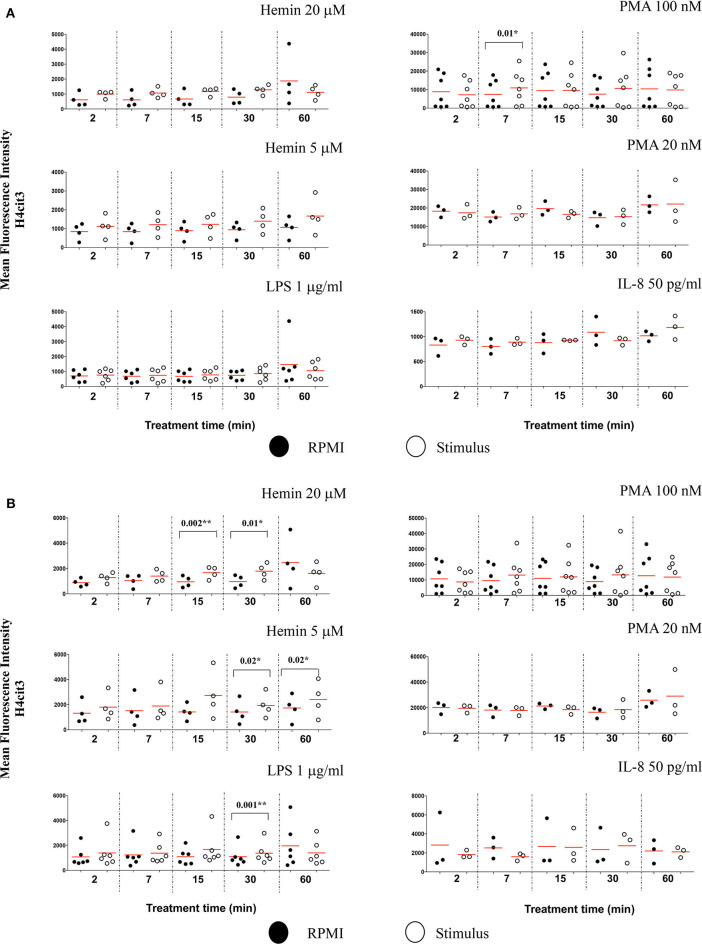
Nuclear decondensation associates with intensity of the H4cit3 signal and is stimulus-dependent. Purified and rested healthy neutrophils were left in RPMI or treated for different lengths of time with NETs inducers as indicated. **(A)** Mean Fluorescence Intensity for H4cit3 in normal nuclei. **(B)** Mean Fluorescence Intensity for H4cit3 in decondensed nuclei. Paired *t*-test was used to compare the response in untreated and treated neutrophils. A variable number of experiments were conducted for each stimulus (*N* = 4 for Hemin 20 μM; *N* = 4 for Hemin 5 μM; *N* = 6 for LPS 1 μg/ml; *N* = 7 for PMA 100 nM; *N* = 3 for PMA 20 nM; *N* = 3 for IL-8 50 pg/ml). Filled symbols, RPMI control; empty symbols, stimulus. Paired *t*-test was used to compare the response in untreated and treated neutrophils. *P < 0.05, **P < 0.01.

We also checked the DNA-MPO Similarity scores in the normal and decondensed nuclei for neutrophils treated with Hemin, LPS, PMA, and IL-8. Confirming the viability of the method, decondensed nuclei had consistently higher DNA-MPO Similarity scores than the normal nuclei, both in untreated and treated samples (Figures S5A,B). We did not confirm higher scores indicating MPO translocation to the nucleus after NETs challenge, neither in the normal nuclei gate nor in the decondensed nuclei gate. Overall, our data indicated either no change, or a decrease in the DNA-MPO co-localization in the treated samples as compared to the untreated control (Figure S5C). We next sought to determine whether following stimulation with the NETs agonists the intracellular levels of H4cit3 or MPO changed (as exemplified in Figure S6A). None of the treatments except PMA 100 nM, increased H4cit3 signal intensity in the normal nuclei (Figure 4A, *P* = 0.01^*^). In the decondensed nuclei 3 stimuli induced significant increase in H4cit3 signal at the longer stimulation. LPS-induced increase was significant at 30 min (*P* = 0.001^*^) suggesting that H4 citrullination might only have a limited contribution to NETs formation by this inducer. Hemin 5 μM caused a notable increase at both 30 and 60 min (with a *P* = 0.02^*^ for both time points) and Hemin 20 μM was efficient at even shorter stimulation (*P* = 0.002^**^ at 15 min and *P* = 0.01^*^ at 30 min). Overall, these data show that while IFC did not confirm DNA-MPO co-localization it did show significant stimulus-dependent changes in H4cit3 levels, consistent with NETs-associated morphological nuclear changes (decondensation).

Terminally differentiated neutrophils do not produce *de novo* MPO following stimulation thus no increase in signal was expected. Stimulus-induced degranulation might explain the mild decrease in the intensity of the MPO signal observed after the NETs treatments (Figure S6B).

### In Early NETosis, Stimulus-Dependent Histone H4 Citrullination Occurs in 1-Lobe Nuclei

The IFC can provide an insight in the relationship between changes in histone H4 citrullination and nuclear segmentation.

**Figure 5:**
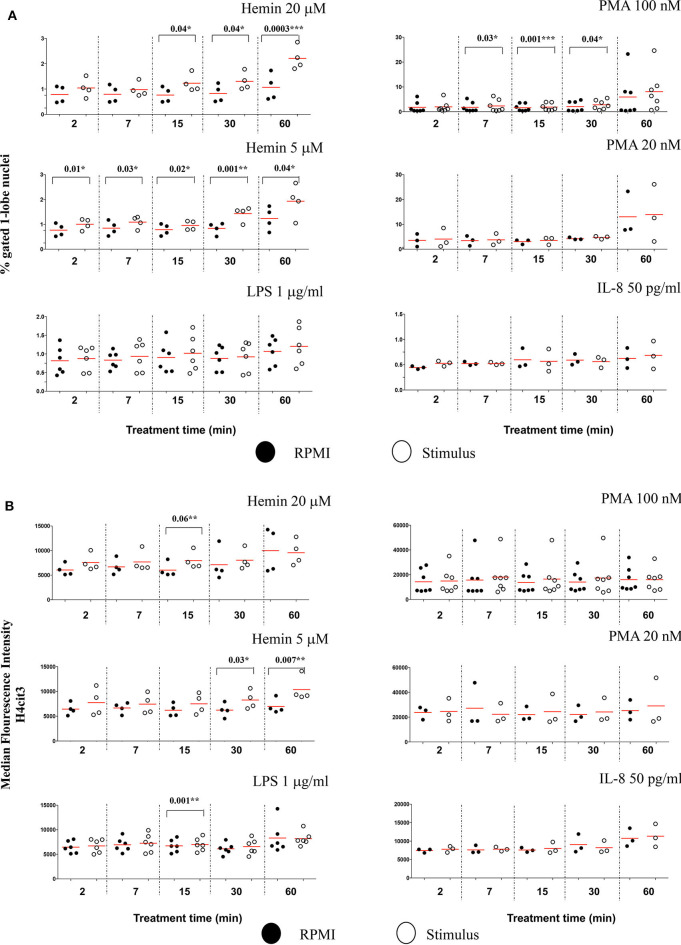
Decrease in neutrophil nuclear segmentation is stimulus- and time-dependent and associates with increased H4 citrullination. **(A)** Purified healthy neutrophils were treated with NETs stimuli for the specified periods of time and percent of neutrophils with 1-lobe nuclei was quantified by using Lobe Count Feature in IDEAS software. **(B)** Median Fluorescence Intensity of the H4cit3 signal in 1-lobe normal nuclei. A variable number of experiments were conducted for each stimulus. Filled symbols, RPMI control; empty symbols, stimulus. Paired *t*-test was used to compare the response in untreated and treated neutrophils. *P < 0.05, **P < 0.01, ***P < 0.001.

The Lobe Count in IDEAS software uses “Symmetry 2, 3, 4” features on the DNA staining channel to count the nuclear lobes, by measuring the number of axis of elongation in a nucleus (for example a nucleus with a single axis will have two lobes and score high in the Symmetry 2 feature), and then bins together the cells that match the criteria. Neutrophils have two to five nuclear segments (i.e., lobes), 40–50% are 3-lobed, about 25%, 2-lobed or 4-lobed, and <10% are 5-lobed. In the untreated samples, we found that generally <1% neutrophils had unsegmented nuclei, and about 40% of the cells were 3-lobed (Figure S7A). After 60 min of treatment with PMA 100 nM, the number of cells with unsegmented nuclei increased by almost 10-fold while the number of those having 3-lobe nuclei decreased discernably (Figure S7B). LPS, IL-8, and PMA 20 nM had no effect on nuclear segmentation at any of the checked time points (Figure 5A). PMA 100 nM and Hemin 20 μM induced significant increases at few of the checked treatment time points, with the first showing the strongest increase after 15 min (*P* = 0.001^**^) and the latter after 60 min (*P* = 0.0003^***^). However, it was the low Hemin concentration that had the most substantial effect on nuclear segmentation. We found that Hemin 5 μM caused a significant increase in cells with 1-lobe nuclei already at 2 min of treatment (*P* = 0.01^*^), up until 60 min (*P* = 0.04^*^), with the biggest change at 30 min (*P* = 0.001^**^) (Figure 5A, Figure S7C). We asked whether an increase in the amount of 1-lobe nuclei associated with an increase in H4cit3 signal in these nuclei. For this we looked at the 1-lobe nuclei amount (Figure 5A) vs. the H4cit3 signal intensity in these nuclei (Figure 5B). For the two Hemin concentrations the increase in H4cit3 intensity generally matched the increase in the 1-lobe nuclei. At the later time points, 30 and 60 min, Hemin 5 μM induced a significant increase in H4cit3 expression (*P* = 0.03^*^ and *P* = 0.007^**^, respectively), strongly suggesting that, for this stimulus, histone citrullination increased in the 1-lobe rather than in the segmented nuclei.

### Imaging Flow Cytometry Offer Additional Analysis Features to Characterize Early NETosis Signature

**Figure 6:**
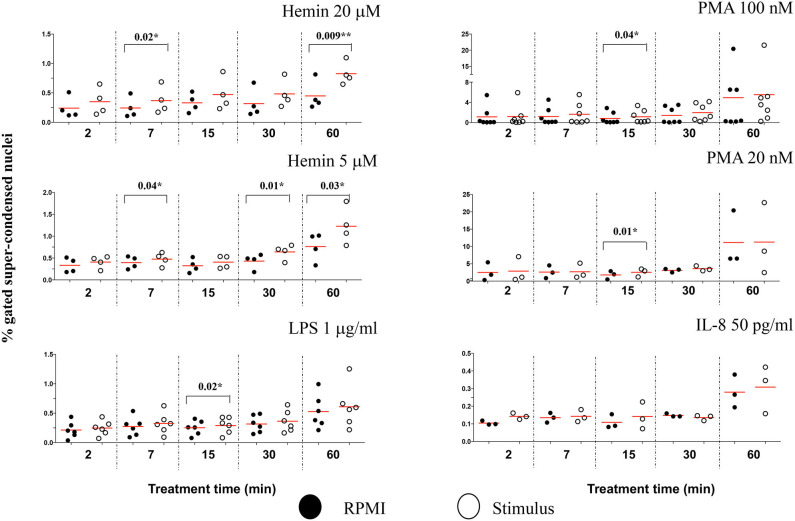
The level of nuclear super-condensation caused by the NETs inducers varies with the stimulus. Purified healthy neutrophils were treated with the NETs stimuli for the shown lengths of time. Percent cells with super-condensed 1-lobe nuclei were identified and quantified by plotting nucleus/whole cell Area vs. Morphology Area (nuclear stain—Hoechst). An example of these analysis features is shown in Figure S8A. A variable number of experiments were conducted for each stimulus. Filled symbols, RPMI control; empty symbols, stimulus. Paired *t*-test was used to compare the response in untreated and treated neutrophils. *P < 0.05, **P < 0.01.

Due to the subjective and semi-quantitative nature of the majority of methodology currently available for NETs studies, specific identification of NETs formation from other cellular processes is still a challenge (14). Our high throughput data suggested that H4 citrullination occurred in nuclei that would lose their organized structures and decondense. Depending on the stimulus, this process was rapid (Hemin) or slower (PMA), and associated with loss in nuclear segmentation. We asked whether IFC could offer another tool for identification of morphological changes in these unsegmented nuclei. The chromatin undergoing strong condensation ahead of the dismantling of the nuclear envelope is recognized as a marker of cellular death (apoptosis). Used together, two analysis features in IDEAS software, dot plot of “Area of nucleus” vs. “Ratio of nuclear by the whole cell areas” can discriminate between levels of nuclear super-condensation in untreated and treated neutrophils (Figure S8A). With the exception of IL-8, all stimuli caused a statistically significant increase in the nuclear super-condensation. LPS, PMA 100 nM, and PMA 20 nM were particularly efficient at 15 min (*P* = 0.02^*^, *P* = 0.04^*^, and *P* = 0.01^*^, respectively). Hemin was efficient as early as 7 min (Hemin 20 μM, *P* = 0.02^*^ and for Hemin 5 μM, *P* = 0.04^*^) and also after 60 min (Hemin 20 μM *P* = 0.009^**^ and for Hemin 5 μM, *P* = 0.03^*^) (Figure 6). Clear nuclear morphological differences were underlined in NETosis (decondensation) and apoptosis (super-condensation). While H4cit3 is a recognized marker of NETosis, Annexin V expression is often used as a marker of apoptosis. We employed conventional flow cytometry to determine whether we can complement our IFC findings for the Hemin-mediated stimulation with a positive detection of a known apoptosis marker, Annexin V. Both Hemin concentrations induced a time-dependent increase in Annexin V expression with a statistical significance at 60 min (Hemin 20 μM, *P* = 0.02^*^ and Hemin 5 μM, *P* = 0.03^*^) (Figures S9B,C). These finding are in agreement with the increase in super-condensation determined at 60 min with the IFC technique. While boosting confidence for accurate detection of NETosis, our findings also offer an option for preliminary detection of apoptosis-related events at the same time.

**Figure 7:**
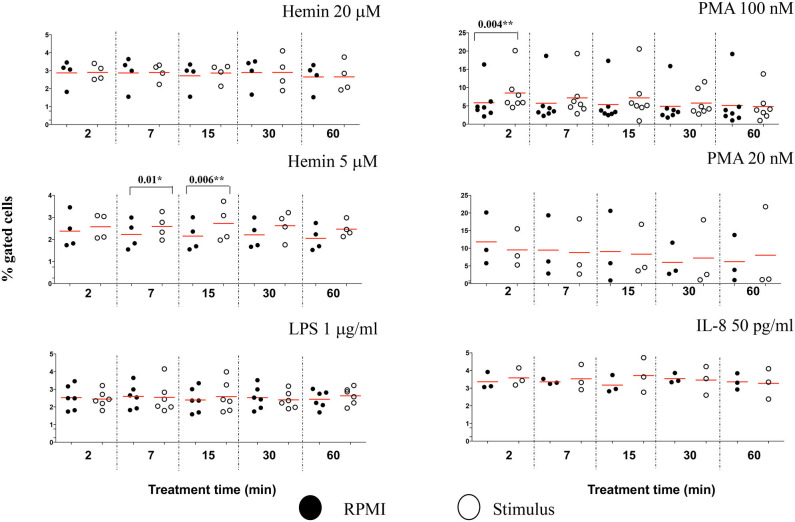
NETs inducers cause minimal damage to neutrophil cell membranes. Following NETs stimulation neutrophils with damaged cell membranes were identified and quantified by plotting Standard Deviation feature on the SSC channel against Modulation in Brightfield, as exemplified in Figure S10A. A variable number of experiments were conducted for each stimulus. Filled symbols, RPMI control; empty symbols, stimulus. Paired *t*-test was used to compare the response in untreated and treated neutrophils. *P < 0.05, **P < 0.01.

To determine whether other cellular changes can be associated with the observed increase in chromatin citrullination, we used “Standard deviation” and “Modulation” features in IDEAS analysis software to assess the cell membrane damage. The Standard Deviation Feature indicates the pixel intensity standard deviation values in the mask and provides an indication of the complexity of an object. Modulation Feature measures the intensity range of an image, and this can be used to quantify image quality and characterize contrast and texture in cells. High values for Standard deviation in darkfield (SSC channel) and for Modulation in brightfield indicate neutrophils with damaged cell membranes (Figure S10A). We showed that PMA 100 nM caused a rapid increased in the percentage of neutrophils with damaged cellular membranes, following just 2 min of treatment (*P* = 0.004^**^), while the lower Hemin concentration efficiently induced cell membrane changes after 7 and 15 min of treatment (*P* = 0.01^*^ and 0.006^**^, respectively) (Figure 7). No significant changes for this analysis parameter were noted during the 60 min of the treatment with any of the used stimuli (Figure S10B). In comparison with healthy neutrophils, IFC competently detected changes cell membrane texture in neutrophils isolated from patients with sickle cell disease at steady state. This environment, known for its high inflammatory potential and high levels of free-circulating heme, was also directly linked with an increased potential for NETs production (15). Thus, while it does appear that NETs-associated cell membrane damage is stimulus-dependent, it might nevertheless be a useful parameter to follow up while characterizing NETosis.

## Discussion

The association of NETs with an increasing number of infectious and non-infectious pathologies calls for more effective and consistent approaches for NETosis assessments. Currently, a lot of the testing is conducted by using fluorescence microscopy to observe extracellular DNA co-localized with NE and/or MPO. Other approaches use quantification of extracellular DNA in cell-free supernatant or examine cellular viability with nuclear counterstains that are impermeant to live cells. Increased plasma levels of cell-free DNA, histones, or NETs associated molecules, such as NE or MPO, have been measured in some inflammatory pathologies (1). Presently, the NETotic capacity of pathological environments is generally assessed indirectly by treating healthy neutrophils with blood-derived fluids from patients and then measuring the NETs response. NETs might also function as biomarkers in certain diseases and provide information regarding the efficacy of a treatment regimen (16). Cell-free DNA or histones are certainly increased in many of the inflammatory diseases, but whether this reflects NETs production or simply the state of dying cells remains unclear. NETs-associated components such as NE or MPO are present in the plasma of individuals with many autoimmune diseases and also in infections, which complicates the specificity of these molecules as biomarkers. In this light there is a great need for alternative methodology for NETs characterization that circumvents the subjective nature of microscopy analysis and offers consistent criteria between studies, reliable for both mechanistic and clinical applications.

We have developed this IFC protocol as a consistent approach for specific, objective and quantitative measurement of a well-established NETs marker (histone H4 citrullination) in combination with several other parameters that can further characterize nuclear and cellular changes associates with NETosis.

In our experience a few steps are critical for the experimental quality and reproducibility of this technique. Due to unavailability of a primarily conjugated antibody for this marker a primary antibody and fluorescent secondary antibody system are used for detection of citrullinated histone 4 in this protocol. For accurate detection and quantification of the H4cit3+ signal, the effectiveness of the blocking buffer is essential to prevent non-specific binding of the secondary antibody (DyLight680). It is thus strongly recommended that the efficiency of the blocking buffer should be confirmed, at the beginning of the experimental work by detecting the DyLight680 antibody signal in the absence of the H4cit3 primary antibody. For data analysis with the IDEAS software, custom masks should be employed because they represent more accurately the region of interest, rather than the default masks. Custom masks for total cell area and nucleus should be first validated for accuracy, by confirming that the masks are indeed masking the region(s) of interest (i.e., not missing parts of the target region or, on the opposite, masking “ghost” regions outside of what is relevant for the features) (17).

Marked variability in the specificity/reactivity of different antibodies targeting histones deimination on different histones has been reported (18). Multiple members of the PAD family mediate conversion of arginine to citrulline on different histones. PAD4 mediates deimination of histone 3 and histone 4, both routinely observed in NETs studies. Previous studies indicate that histone citrullination might be stimulus-depended. For example, PMA has been reported to induce histone H3 deimination in mouse (19). In humans, H3 citrullination might not be required for PMA-induced NETs formation after a 2-h long treatment (18). PAD4-mediated citrullination following shorter stimulation is less understood.

The protocol we have developed is sensitive enough to detect the H4cit3 marker in neutrophils treated for under 1 h with various NETs inducers. Our data show that H4cit3 site is relevant for PMA-induced NETosis, at both low and high concentrations, within the 1st hour of stimulation (Figure 2 and Table 2). However, while pharmacological stimuli are convenient tools for research, they may not be so relevant and translatable for clinical purposes. Hence, in our study we included LPS, IL-8, and Hemin, products that are relevant clinically. We showed that the H4cit3 does not appear to be majorly involved in early NETosis induced with LPS and IL-8, but appears to strongly contribute to NETosis mediated by Hemin (Figures 2D, 4B).

**Table 2:** NETs stimuli time-dependent response efficiency.

**Parameters**	**Hemin 20 μM**	**Hemin 5 μM**	**LPS 1 μg/ml**	**PMA 100 nM**	**PMA 20 nM**	**IL-8 50 pg/ml**
**2 min**						
Decondensed nuclei (%)					^*^	
H4cit3 in normal nuclei (%)					^**^	
H4cit3 in decondensed nuclei (%)					^**^	
H4cit3 in normal nuclei (MFI)					^**^	
H4cit3 in decondensed nuclei (MFI)					^**^	
1-lobe normal nuclei (%)					^*^	
Super-condensed normal nuclei (%)						
Damaged membrane and normal nuclei (%)						
**7 min**						
Decondensed nuclei (%)				^*^	^**^	
H4cit3 in normal nuclei (%)					^**^	
H4cit3 in decondensed nuclei (%)				^*^	^*^	
H4cit3 in normal nuclei (MFI)					^*^	
H4cit3 in decondensed nuclei (MFI)						
1-lobe normal nuclei (%)						
Super-condensed normal nuclei (%)						
Damaged membrane and normal nuclei (%)						
**15 min**						
Decondensed nuclei (%)				^*^	^**^	
H4cit3 in normal nuclei (%)					^**^	
H4cit3 in decondensed nuclei (%)					^*^	
H4cit3 in normal nuclei (MFI)					^*^	
H4cit3 in decondensed nuclei (MFI)					^*^	
1-lobe normal nuclei (%)					^**^	
Super-condensed normal nuclei (%)					^**^	
Damaged membrane and normal nuclei (%)						
**30 min**						
Decondensed nuclei (%)				^*^	^**^	
H4cit3 in normal nuclei (%)					^*^	
H4cit3 in decondensed nuclei (%)				^*^	^**^	
H4cit3 in normal nuclei (MFI)					^*^	
H4cit3 in decondensed nuclei (MFI)				^*^	^*^	
1-lobe normal nuclei (%)					^**^	
Super-condensed normal nuclei (%)					^**^	
Damaged membrane and normal nuclei (%)						
**60 min**						
Decondensed nuclei (%)				^*^	^***^	
H4cit3 in normal nuclei (%)						
H4cit3 in decondensed nuclei (%)				^*^	^***^	
H4cit3 in normal nuclei (MFI)					^**^	
H4cit3 in decondensed nuclei (MFI)					^**^	
1-lobe normal nuclei (%)					^*^	
Super-condensed normal nuclei (%)						
Damaged membrane and normal nuclei (%)						

*Anova with Dunnett's Multiple Comparison Test was used to compare stimuli response to the control group (RPMI) at each of the tested time points. (RPMI, N = 27; Hemin 20 μM, N = 4; Hemin 5 μM, N = 4; LPS 1 μg/ml, N = 6; PMA 100 nM, N = 7; PMA 20 nM, N = 3; IL-8 50 pg/ml, N = 3)*.

* P < 0.05,

** P < 0.01,

*** *P < 0.001*.

Because of its potential contribution to initiation and/or progression of pathological conditions, infection-related NETosis has been studied extensively in both mouse and human models (20, 21). Newer data shows that that PAD4-mediated citrullination of histone 3 is required for LPS-induced NETs formation (22, 23). Here, we showed that PAD-4-mediated H4cit3, did not appear to contribute notably to LPS-mediated NETosis. Nor was this site involved extensively in IL-8 mediated NETosis. IL-8 is an early marker of infection and its production is increased under LPS stimulation (24). Our observations suggest that NETs production under infectious and non-infectious conditions appear to follow distinct paths, likely from the initiation of the process.

Additionally, the Hemin-related results of our study might be of particular interest in chronic hemolytic conditions, where cell-free heme (Hemin), an oxidized product of the hemoglobin, is released from erythrocytes. Heme is a DAMP molecule and has been shown to be an effective trigger of NETosis (25) contributing to the sterile inflammatory condition. Our data show that IFC can confidently detect and quantify H4cit3 even after short exposure to Hemin.

As more evidence link NETs production with an increasing number of pathologies, it is becoming clearer that not all NETs are the same. Different stimuli have different potencies and initiation mechanisms, and the formed DNA-scaffolded strands appear to carry distinct cargo proteins. This variability, compounded by the high potential for non-specific *in vitro* activation of neutrophils, can hinder the development of reliable detection and quantification methods.

We aimed at developing a reliable detection method that would identify the earlier events, as neutrophils prepare for NETs production in response to physiological stimuli (such as cytokines—IL-8 or hemolysis product—Hemin). With our induction and detection methodology, the H4cit3 response for all tested stimuli was quantifiable, either in normal or decondensed nuclei. PMA and both Hemin concentrations were potent agonists that induced strong NETosis-related early nuclear changes; at the same time LPS and IL-8 had limited effects (Figures 2, 4).

This methodology offers advantages for both research and clinical projects seeking reliable detection and quantification of markers involved in early NETosis. Our method does not require any additional information (e.g., the release of reactive oxygen species) to deliver quantifiable data on histone 4 citrullination and nuclear decondensation. Neutrophils are minimally handled (1-step isolation and no plating required) to reduce exposure to unspecific activation signals. This technique distinguishes and quantifies a known NETs marker (e.g., H4 histone citrullination) in addition to nuclear decondensation, in whole neutrophils with an intact cellular membrane, prior to the DNA extrusion. If required, this technique also allows quantitative evaluation and/or by imagery of DNA-free neutrophils as it allows specific gating of CD66b+Hoechst- cells. This can be particularly suitable for studies interested to look as NET-ing neutrophils as potential biomarkers for diseases.

Our method quantified NETosis-related events for potent and less potent stimuli. However, with the limited data from the LPS and IL-8 stimuli, it also emphasized that detection limitations might be associated with some stimuli.

## Data Availability Statement

The datasets generated for this study are available on request to the corresponding author.

## Ethics Statement

The NHLBI Institutional Review Board approved the study that was conducted in accordance with the ethical principles stated in the Declaration of Helsinki. The patients/participants provided their written informed consent to participate in this study.

## Author Contributions

EB and VD wrote the manuscript. EB developed the staining technique. VD established the Imaging Flow Cytometry analysis parameters. LM performed experimental work and revised the manuscript. ST supervised the development of the project and revised the manuscript. All authors contributed to the article and approved the submitted version.

## Conflict of Interest

The authors declare that the research was conducted in the absence of any commercial or financial relationships that could be construed as a potential conflict of interest.
