# Supplementary material for: Detection and Quantification of Histone H4 Citrullination in Early NETosis With Image Flow Cytometry Version 4
Source: Front Immunol. 2020 Jul 16;11:1335. doi: 10.3389/fimmu.2020.01335 (PMC7378400; doi:10.3389/fimmu.2020.01335)
Supplement: Supplementary file 1 [file Data_Sheet_1.docx]

**Supplemental Methods**

*NETosis Staining Technique for Imaging Flow Cytometry*

We have developed a specific histone citrullination staining protocol using a primary antibody from Millipore (H4cit3), and a secondary antibody conjugated with DyLight680 fluorochrome.

1. In the 1.5 ml Eppendorf tubes re-suspend fixed and thoroughly washed cells in 100 μl 2% BSA containing CD66b-PE (5 μl/test), mix vigorously for at least 5 times and incubate in the dark for 20 minutes at room temperature.
2. Re-suspend in 300 μl BD Cytofix/Cytoperm, mix by pipetting up and down at least 5 times, and incubate 15 minutes in the dark at room temperature.
3. Re-suspend pellet in 500 μl of blocking buffer (3% BSA in 1xDPBS, no calcium, no magnesium, + 0.2% porcine skin gelatin), mix by pipetting up and down at least 5 times, incubate overnight at 4°C.
4. Prepare unstained control at this point.
5. Re-suspend in 100 μl of 2% BSA containing anti-histone H4 citrulline 3 (H4cit3) primary antibody. Pipet up and down at least 10 times and incubate overnight at 4°C.
6. Re-suspend pellet in 100 μl of 2% BSA containing secondary antibody DyLight 680, at 1:1000 dilution. Pipet up and down at least 10 times and incubate for 30 minutes in the dark at room temperature.
7. Re-suspend in 200 μl of 2% BSA containing Hoechst diluted at 1:1000. Mix thoroughly and incubate for 15 minutes in the dark at room temperature.
8. Re-suspend in 50 μl WB for ImageStream (Imaging Flow cytometer) acquisition.

*NOTE: Thoroughly wash pellet between the steps at least 3 times with 500 μl wash buffer (2% BSA in 1xDPBS, no calcium, no magnesium, + 2mM EDTA).*

*Imaging Flow Cytometry Acquisition and Data Analysis.*

Fixed and stained samples can be acquired within 7 days from the completion of the staining step without changes in the quality of the signal. Acquisition of at least 20,000 cells (events) per test should be considered to provide statistical power and robustness of the data in analyzed subset populations. Acquisition should be conducted while cells are in focus (quality of the focus should be monitored and adjusted, if needed, during acquisition). For more detailed information about how to run samples on ImageStream, please refer to the Imagestream User’s Manual in the Amnis Customer Portal - MilliporeSigma webpage.

*Imaging Flow Cytometer Acquisition*

1. Within the INSPIRE software, create an acquisition template to be used with all experimental repeats, keeping the same settings for laser powers, magnification and number of events acquired. Item 5 (below) is required for each new acquisition; the remaining items are covered in an established and saved template.
2. Under the “Illumination” tab turn on the 785 laser (side scatter laser – SSC) and all other lasers to be used in the experiment, based on the fluorochromes panel choice (a chart showing the laser excitation wavelength and its corresponding dyes is available in the company’s website).
3. Under the “Magnification” section adjust magnification to 60X.
4. Under the “Fluidics” section slide the Speed/Sensitivity bar to the left (to ensure acquisition with low speed and high sensitivity).
5. Under “File Acquisition” create a new folder to save the acquired files (.rif – *raw image file* extension).
6. Load a sample stained with all the colors expected to yield the brightest signals for all the dyes used. Adjust the laser voltages based on the maximum of fluorescence of each fluorochrome according to their laser excitation.

*NOTE: When setting the channel-specific laser power, the optimal voltage of the laser is the one that does not saturate the detectors (less than 4096 pixels in the generated image) and also promotes a clear distinction between negative and positive populations. A ‘Raw Max Pixel’ feature of each of the used channels should be applied. This feature provides the largest value of the pixels contained in the input mask.*

1. Once the laser voltages are set, “Return” the sample and set it aside to re-acquire it later after the single-color compensation control tubes have been acquired.
2. Acquire the compensation controls and generate a compensation matrix that can be applied to multiple experiments. The customers portal on the Amnis ImageStream website provides detailed instructions of how to run compensation matrix using the Wizard in the INSPIRE software.

*NOTE: A compensation matrix has to be applied to every acquired .rif file when first opened for analysis (even when a compensation matrix was also applied during the acquisition of that file with INSPIRE).*

1. Create a histogram of Area of the Brightfield (BF) channel and gate for events to be inside the range of 50µm^2^ to 600µm^2^ area to exclude debris, small particles and aggregates.
2. From the gate above, create a histogram using the gradient RMS (root mean square) feature of the BF (Channels 1 or 9), to select the cells in focus. Focused cells are usually within the score of 40 and above, but this can be adjusted by visualization of the cells to set the gate properly.
3. Set the “Acquisition” tab to acquire 20,000 single and focused cells (first and second histograms created, respectively).
4. Save this template to be used for subsequent acquisitions.
5. Type the name of the sample into the “Acquisition” tab.
6. “Load” the sample, confirm cells are centered and in focus and click “Acquire”.

*Imaging Flow Cytometer Analysis*

1. In the IDEAS software, open the .rif extension file to be analyzed.
2. Apply the compensation matrix to be used.
3. Create a dot plot of area of the BF image (M01 – default mask) versus side scatter (SSC – channel 6 or channel 9).

*NOTE: Single neutrophils with high SSC and relatively big cellular area will appear as a distinct clustered population. Check a few of the single cells to define the boundaries of the singlets gate and exclude debris and mononuclear cells.*

1. Select the focused cells by using the focus quality feature in brightfield, Gradient RMS (Root Mean Square).
2. Create a dot plot of signal intensity for granulocyte-specific marker CD66b versus Hoechst and gate double positive events (CD66b+Hoechst+) for further analysis.
3. Gating strategy for neutrophils identification by using IDEAS software is exemplified below. **A)** Single cells identified with two-parameter dot-plot of area of Brightfield (BF - X axis) versus Intensity of Side Scatter on Channel 6 (SSC - Y axis); **B)** Histogram selecting focused singlets by using Gradient RMS (Root Mean Square) in Brightfield; **C)** Neutrophils (CD66b+Hoechst+, in green) identified from singlet focused cells in a two-parameter dot-plot of fluorescence of Hoechst on Channel 7 (X axis) and CD66b on Channel 3 (Y axis). Representative hand-picked cells in all gates (including CD66b+Hoechst-, in yellow; CD66b-Hoechst+, in purple) are shown on sides. The intensity values were artificially gained for the purpose of better visibility and do not affect fluorescence intensity data analysis.


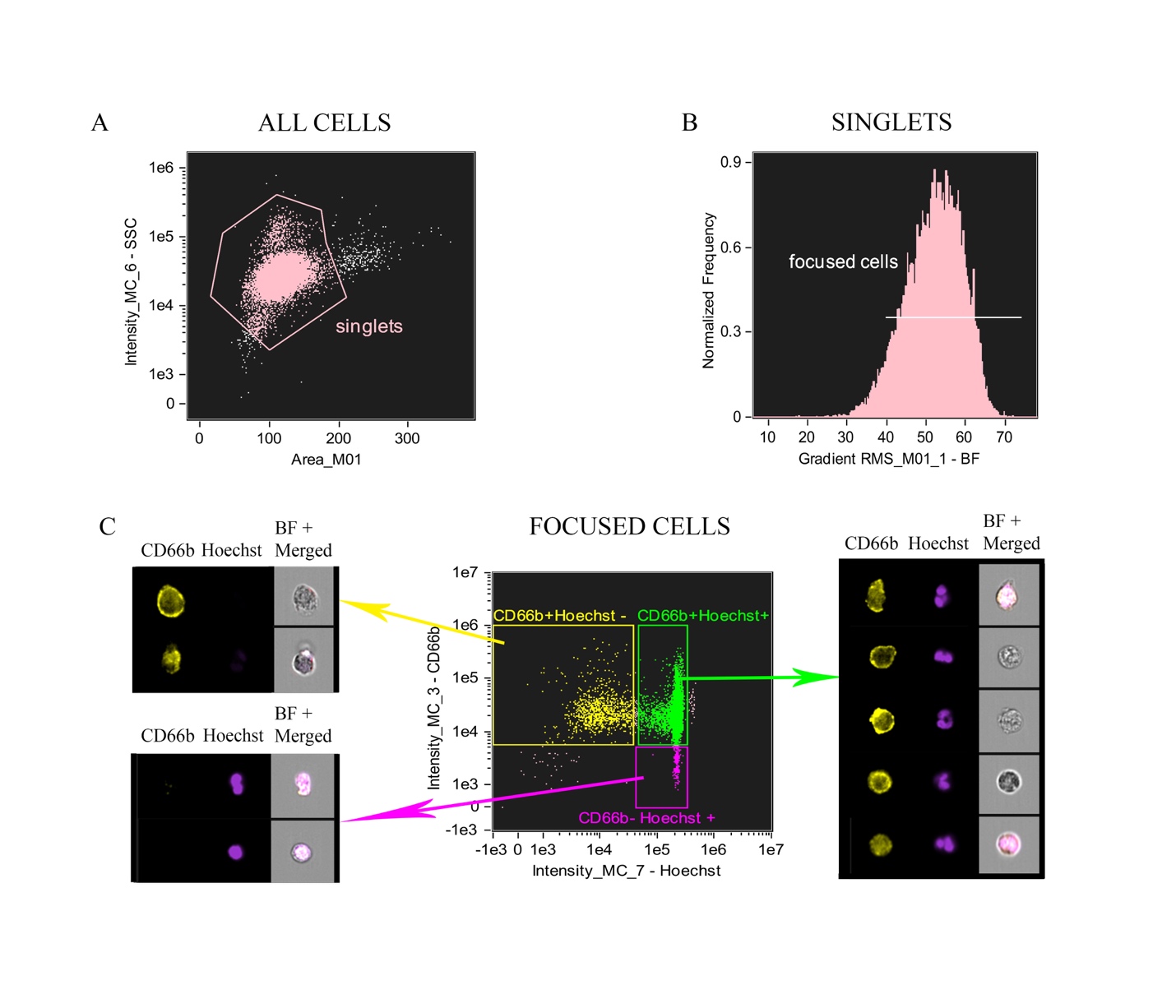


**Supplemental Figures and Tables**

**
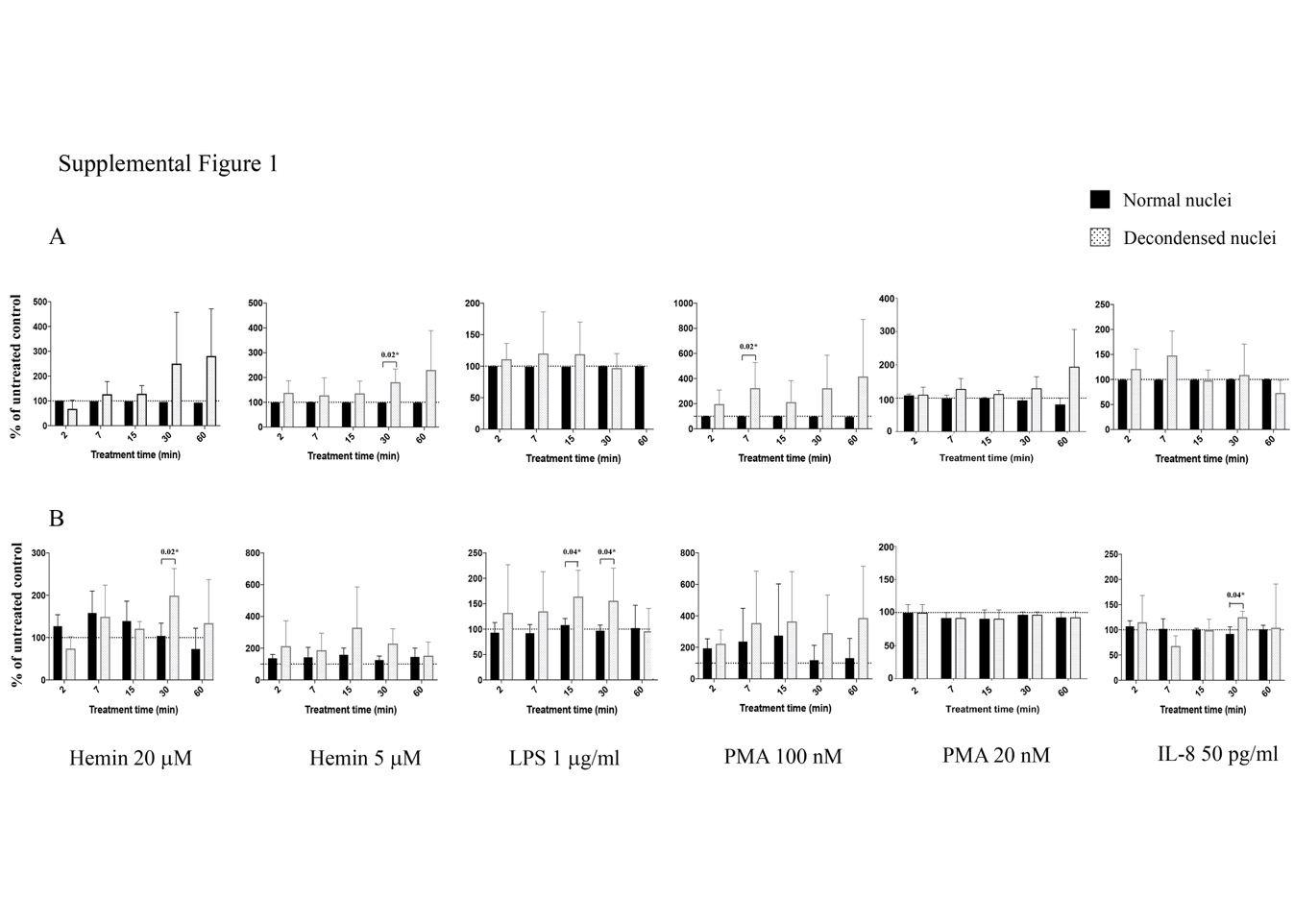
**

**
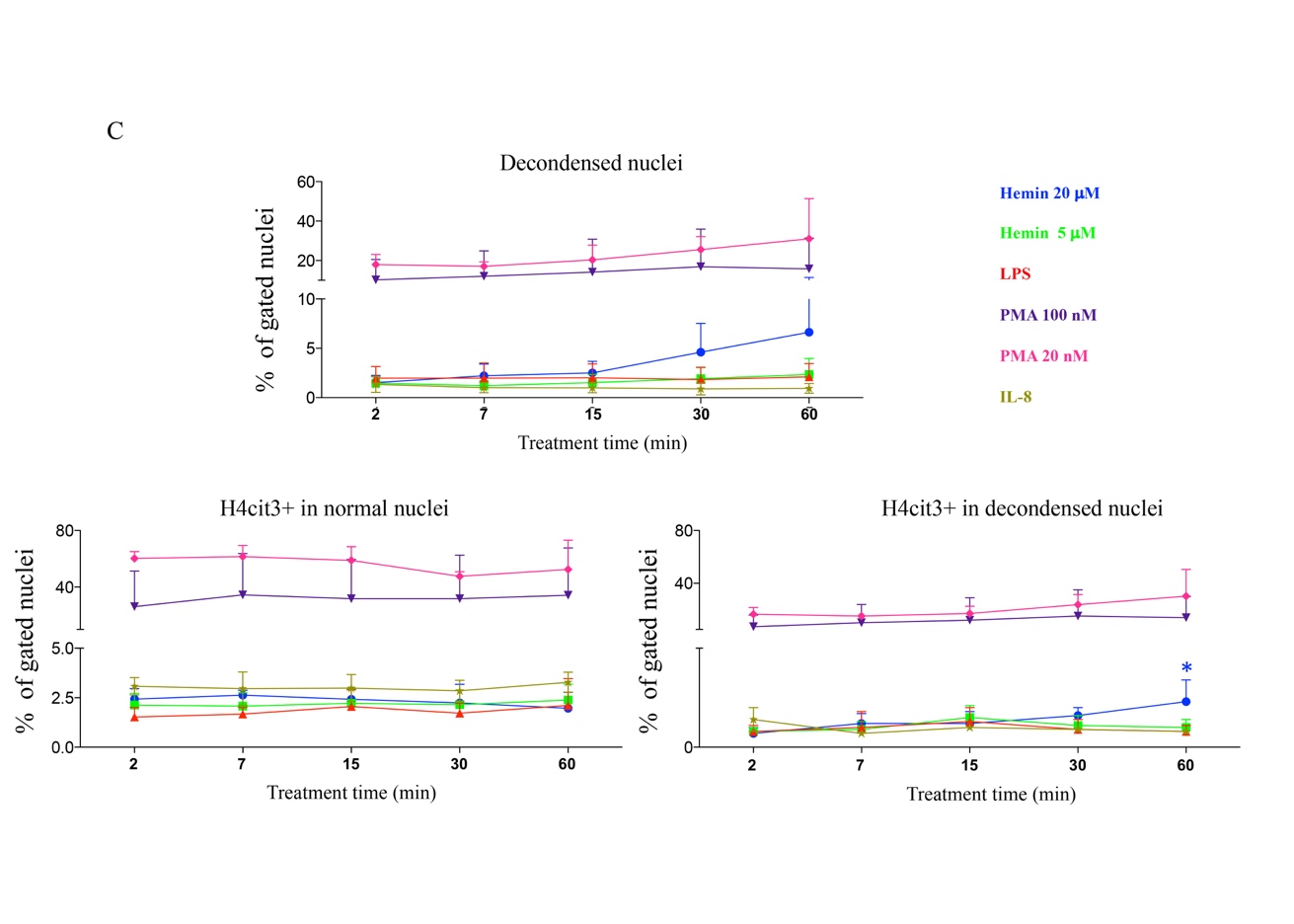
**

**Supplemental Figure 1. Markers of NETosis can be identified following short stimulation and are stimulus-dependent.** Purified healthy neutrophils were treated with the NETs stimuli as indicated. **A**. Summary of changes in nuclear decondensation following treatments. **B.** Post-treatments changes in decondensed nuclei positive for H4cit3. Unpaired t test was used to compare normal nuclei and decondensed nuclei. Data shown as percent change from the RPMI sample (i.e. untreated control) with the average value of all repeats per stimulus ± S.D. presented (100 = no change from the untreated control). **C**. Changes in the percentage of neutrophils with decondensed nuclei and H4cit3+ normal and decondensed nuclei during the length of the treatment with the NETs stimuli. Anova was used to compare 2 minutes with each of the subsequent treatment times for each stimulus. A variable number of experiments were conducted for each stimulus. (N=4 for Hemin 20 μM; N=4 for Hemin 5 μM; N=6 for LPS 1 μg/ml; N=7 for PMA 100 nM; N=3 for PMA 20 nM; N=3 for IL-8 50 pg/ml). *P<0.05.


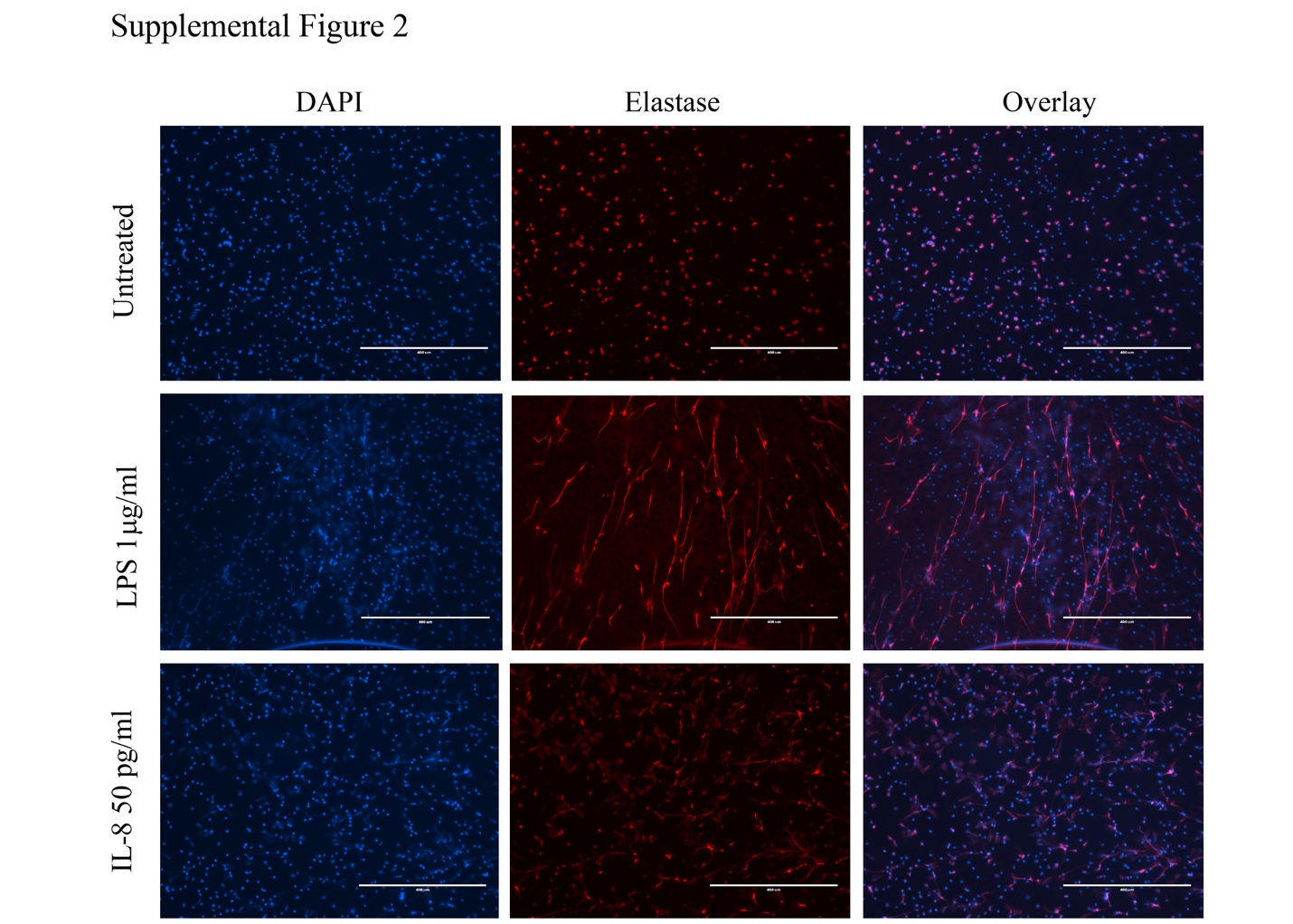


**Supplemental Figure 2. Neutrophils produce NETS following 60 minutes treatment with LPS and IL-8.** Representative immunofluorescence microscopy images of neutrophils confirming production of DNA-Elastase strands when treated with LPS 1 μg/ml and IL-8 50 pg/ml. Briefly, purified healthy neutrophils were plated in plated in poly-L-Lysine coated glass chambers and allowed to rest for 30 minutes prior to the stimulation step. NETs were visualized with primary antibody rabbit polyclonal anti-neutrophil elastase (anti-NE, Abcam, cat # ab21595) and a secondary goat anti-rabbit IgG Alexa Fluor-594 cat #A11037 (Thermo Fisher Scientific, Life Technologies) and DAPI nuclear staining, with an Evos All-in-One Digital Microscope (Fisher Scientific, Life Technologies). Bar is 400 μm.


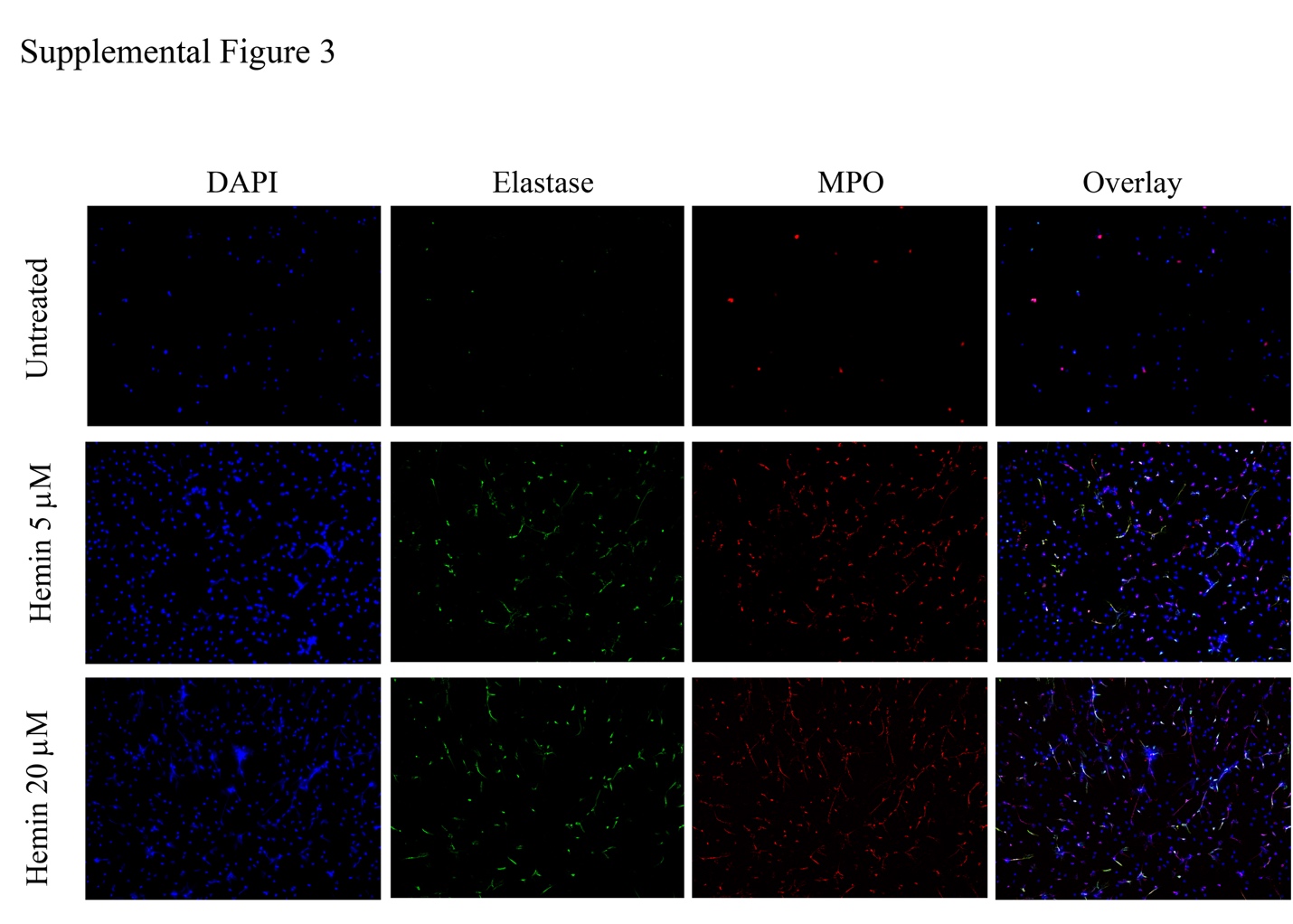


**Supplemental Figure 3.** **Neutrophils produce NETS following 60 minutes treatment with Hemin.** Representative immunofluorescence microscopy images of neutrophils confirming production of DNA-Elastase strands when treated with Hemin. Rested healthy neutrophils, plated in poly-L-Lysine coated glass chambers were treated for 60 minutes with two Hemin concentrations, 5 μM or 20 μM. NETs were visualized with primary antibody rabbit polyclonal anti-neutrophil elastase (anti-NE, Abcam, cat # ab21595) and a secondary goat anti-rabbit IgG Alexa Fluor 488 cat #A11008 (Thermo Fisher Scientific, Life Technologies), anti-human MPO, PE conjugated (clone REA491, Miltenyi Biotec, cat # 130-107-178) and DAPI nuclear staining. Slides were visualized with a BZ-X710 All-in-One Fluorescence Microscope (Keyence, Osaka, Japan).

**
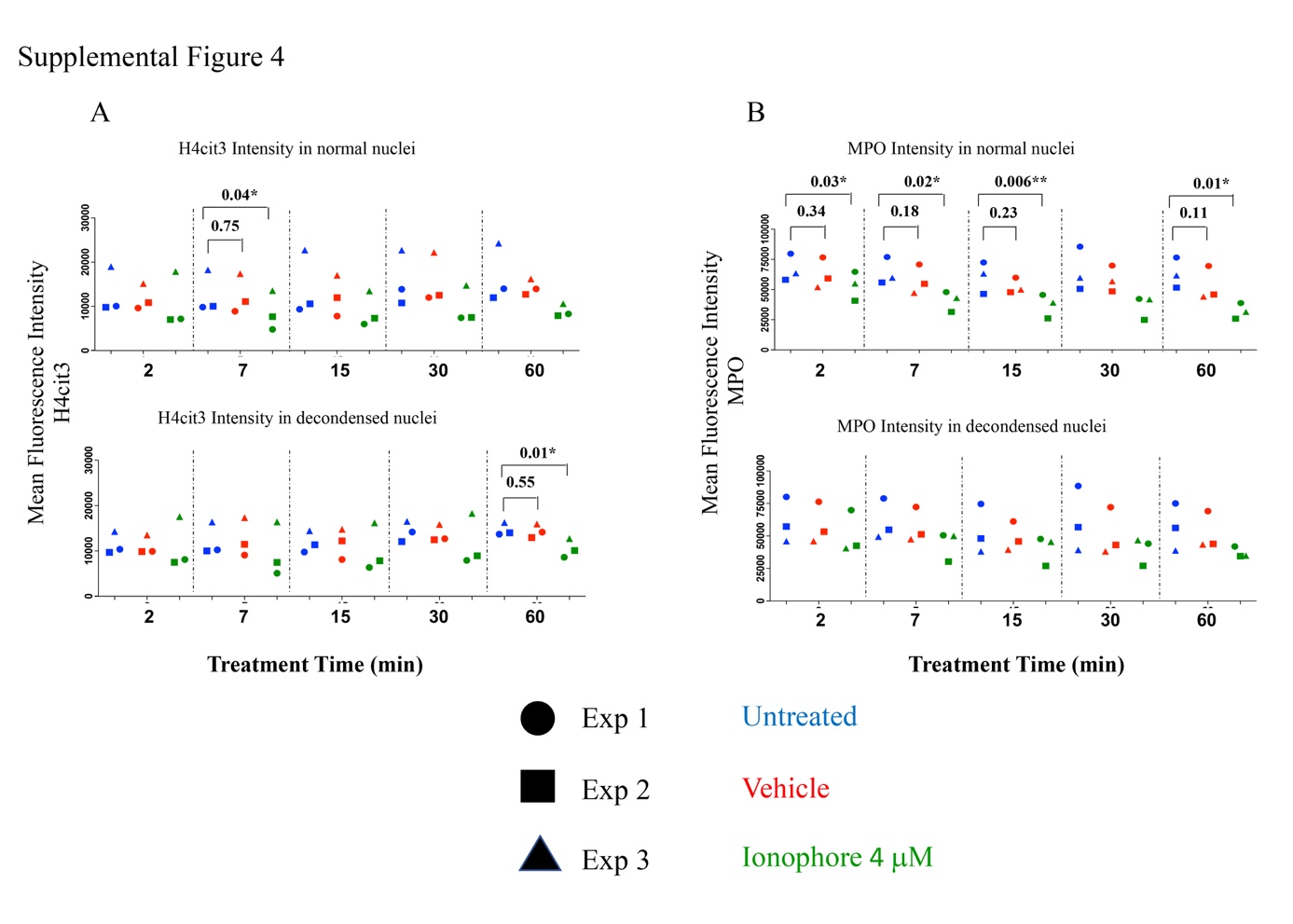
**

**Supplemental Figure 4. Calcium ionophore treatment causes minimal changes in Mean Fluorescence Intensities (MFIs) for H4cit3 and MPO in both normal and decondensed neutrophil nuclei.** Neutrophils purified from 3 healthy donors were left in DPBS (blue), or treated for the indicated times with vehicle only, DMSO (red) or A23187 4μM (green). **A.** H4cit3 MFI’s in normal and decondensed nuclei. **B**. MPO MFI’s in normal and decondensed nuclei. Paired t test was used to compare untreated with vehicle and ionophore 4 μM, respectively, at each time point. *P<0.05, **P<0.01.


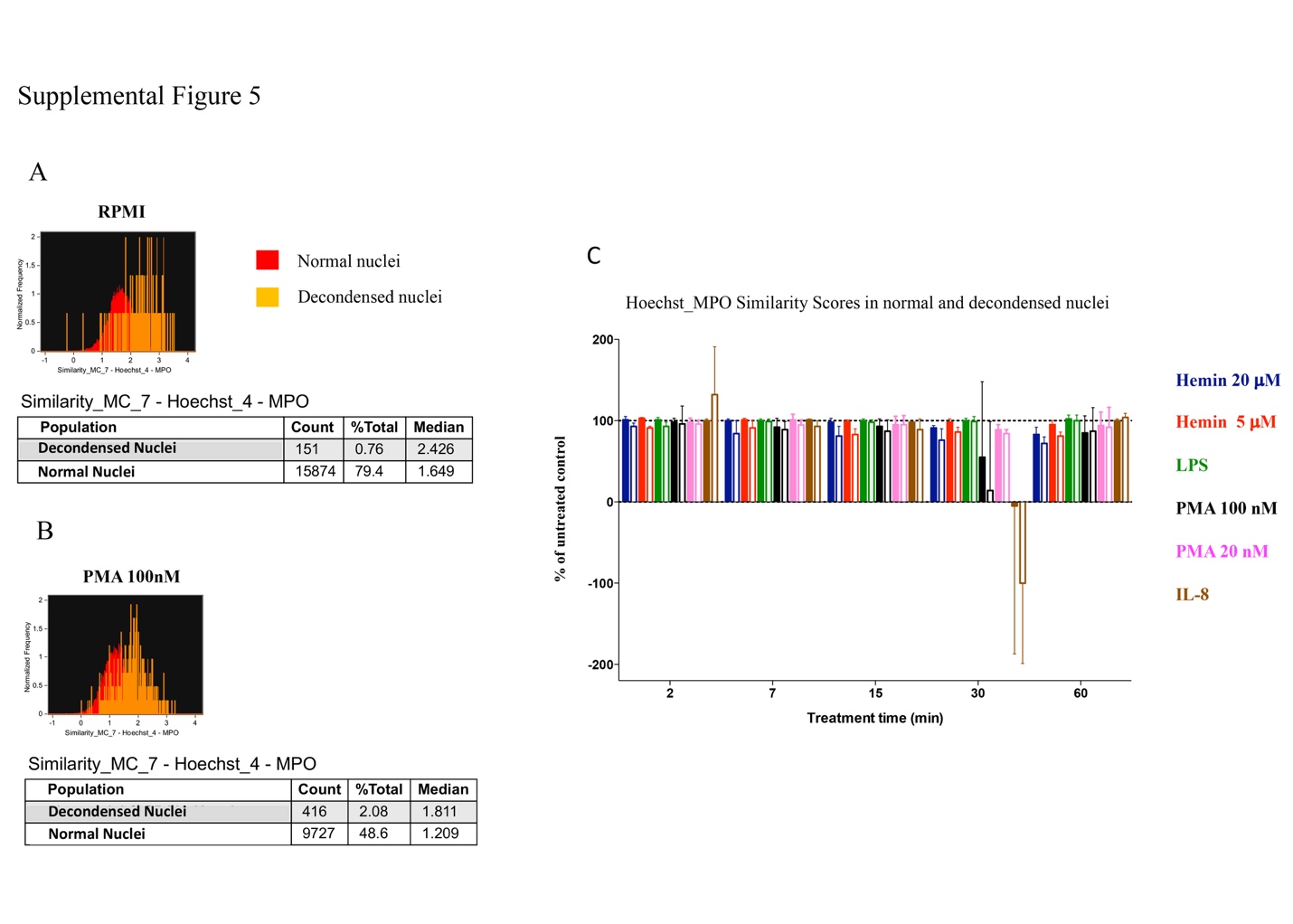


**Supplemental Figure 5. Short-term treatment with NETs inducers does not induce MPO co-localization with the nuclear compartment. A**. Similarity score DNA (Hoechst stain) and MPO normal nuclei (red) and decondensed nuclei (tangerine) in neutrophils incubated for 30 minutes in RPMI. **B**. Similarity score DNA (Hoechst stain) and MPO in neutrophils treated for the same length of time with PMA 100nM. **C**. Summary of changes in DNA_MPO similarity scores following treatment with stimuli at various lengths of time as indicated. Data shown as percent change from the RPMI sample (i.e. untreated control) with the average value of all repeats per stimulus ± S.D. presented (100 = no change from the untreated control). Filled columns – normal nuclei; empty columns – decondensed nuclei.


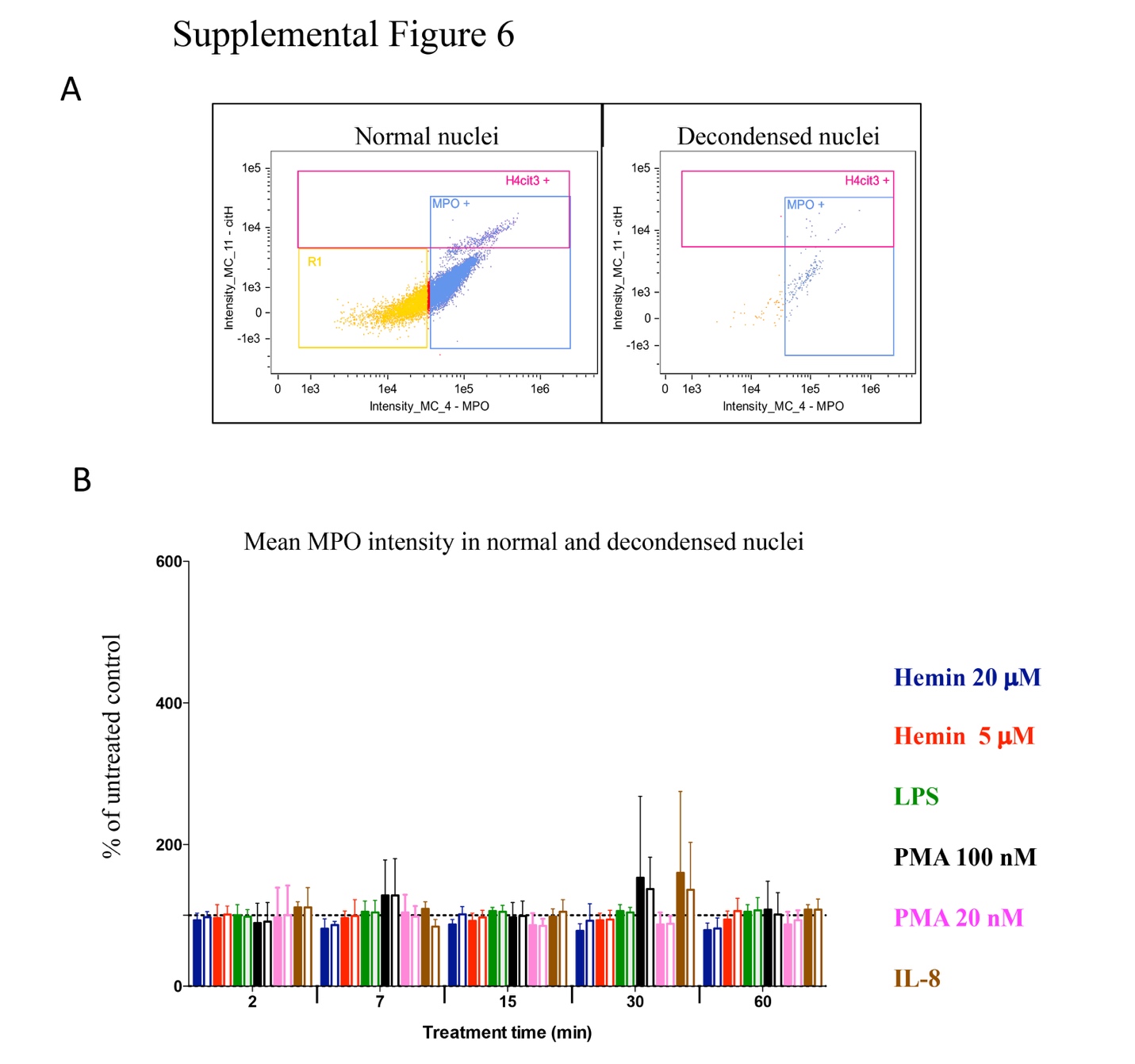


**Supplemental Figure 6.** **Nuclear decondensation associates with intensity of the H4cit3 signal, but not that of the MPO.** **A**. Gating example for quantification of signal intensity for MPO (blue) and H4cit3 (pink) in normal and decondensed nuclei. R1 designates the control MPO unstained gate as identified from the unstained control sample. Fluorescence minus one (FMO) control was used for correct positive gating on the MPO channel. **B.** Summary of changes in the mean MPO intensity following NETs treatment. Change in response to treatments was calculated as percent from the RPMI sample (i.e. untreated control); average value of all repeats per stimulus ± S.D. is presented (100 = no change from the untreated control). Filled columns – normal nuclei; empty columns – decondensed nuclei.

**
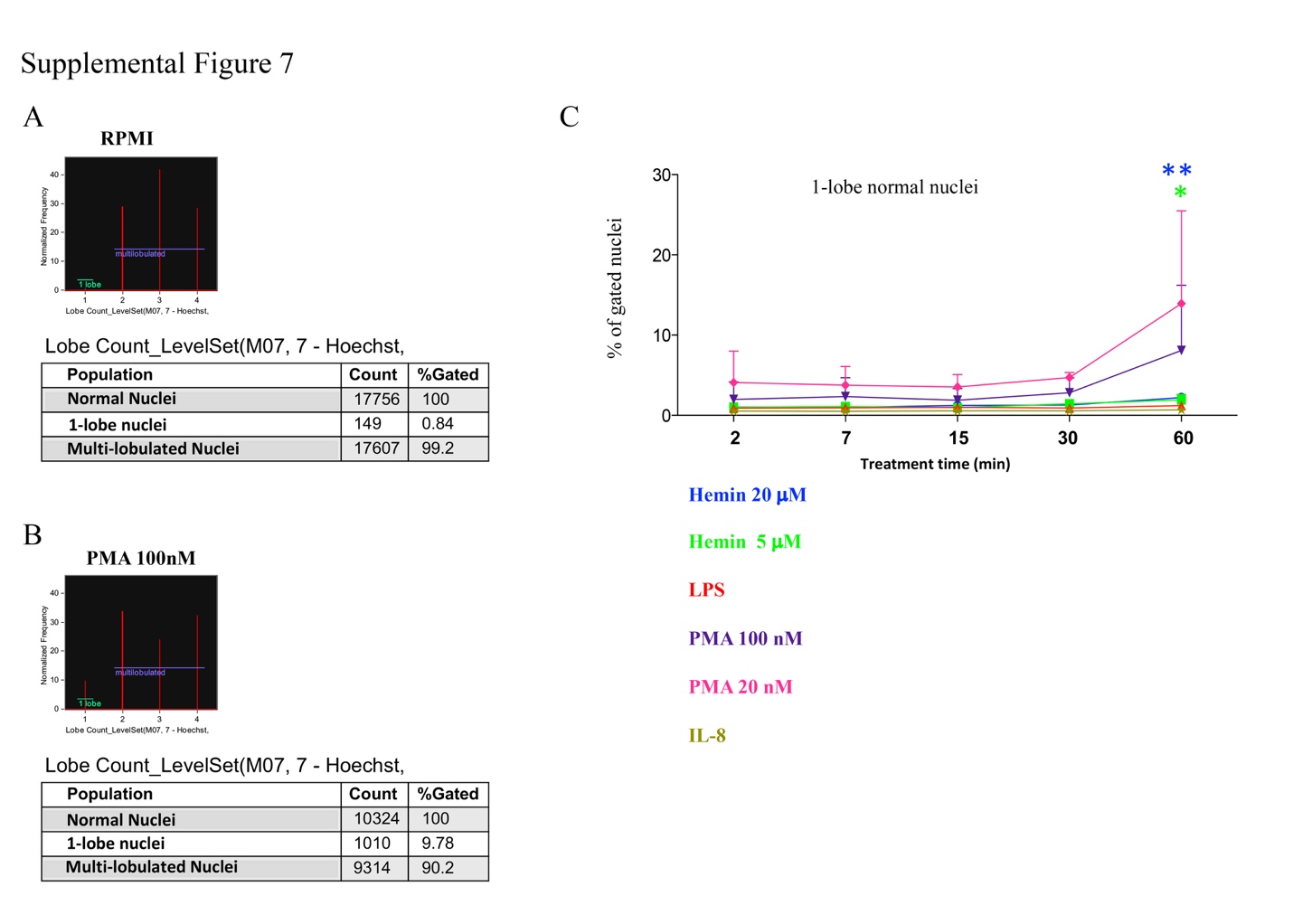
**

**Supplemental Figure 7. Decrease in nuclear segmentation in healthy human neutrophils is slow and stimulus-dependent.** Example of nuclear segmentation quantified in healthy neutrophils incubated for 60 minutes in RPMI **(A)** or PMA 100nM **(B)** by using Lobe Count Feature in IDEAS software. **C**. Percent of neutrophils with 1-lobe normal nuclei gated over the 5 tested treatment time points. Anova compared to the 2 minutes treatment for each stimulus. *P<0.05, **P<0.01.

**
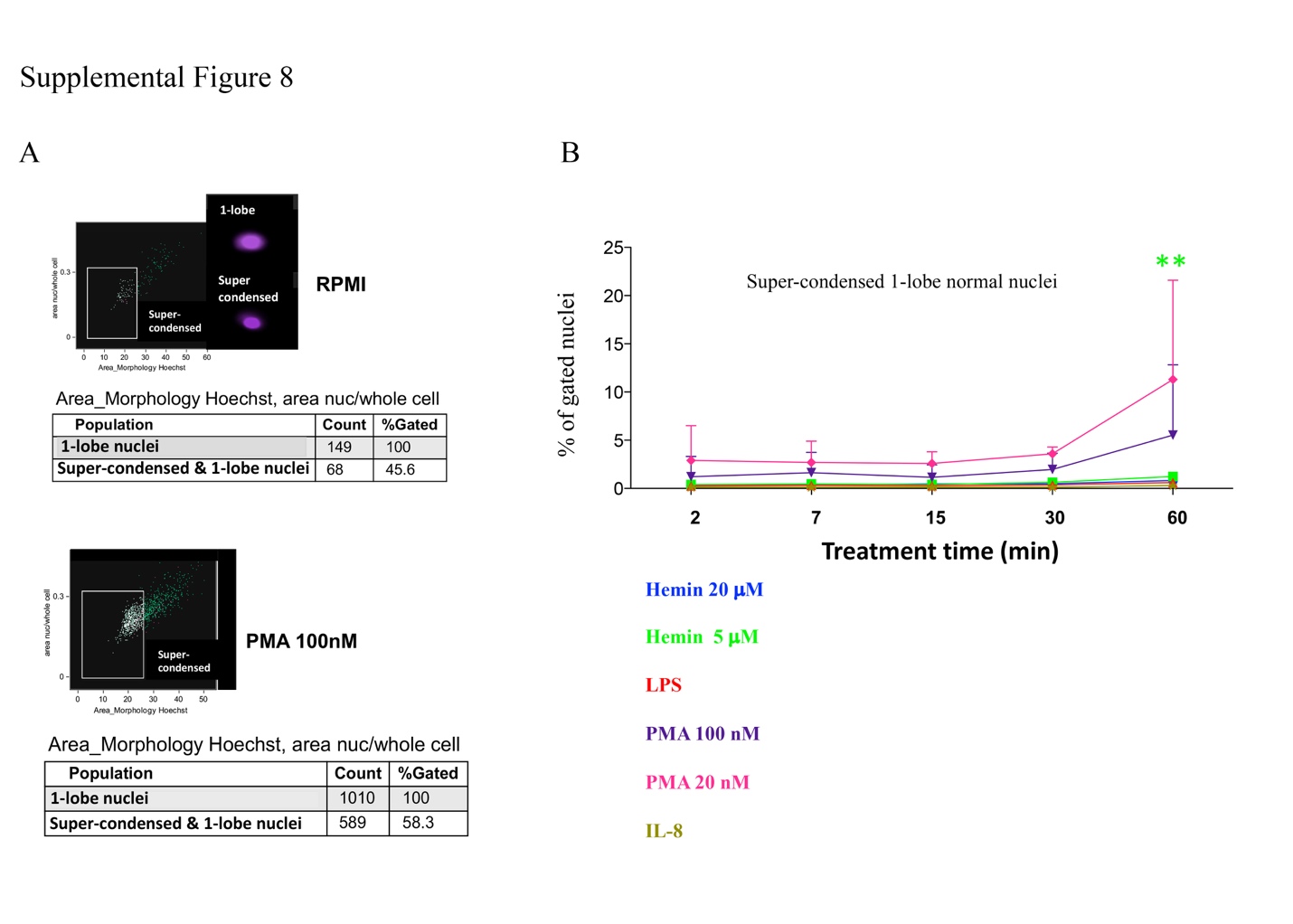
**

**Supplemental Figure 8. NETs stimuli cause an Increase nuclear super-condensation in a stimulus- and time-dependent manner. A.** Example of nuclear super-condensation in normal nuclei of neutrophils incubated for 60 min in RPMI or PMA 100nM. **B**. Percent of neutrophils with super-condensed 1-lobe normal nuclei gated over the 5 tested treatment time points. Anova compared to the 2 minutes treatment for each stimulus. **P<0.01.

**
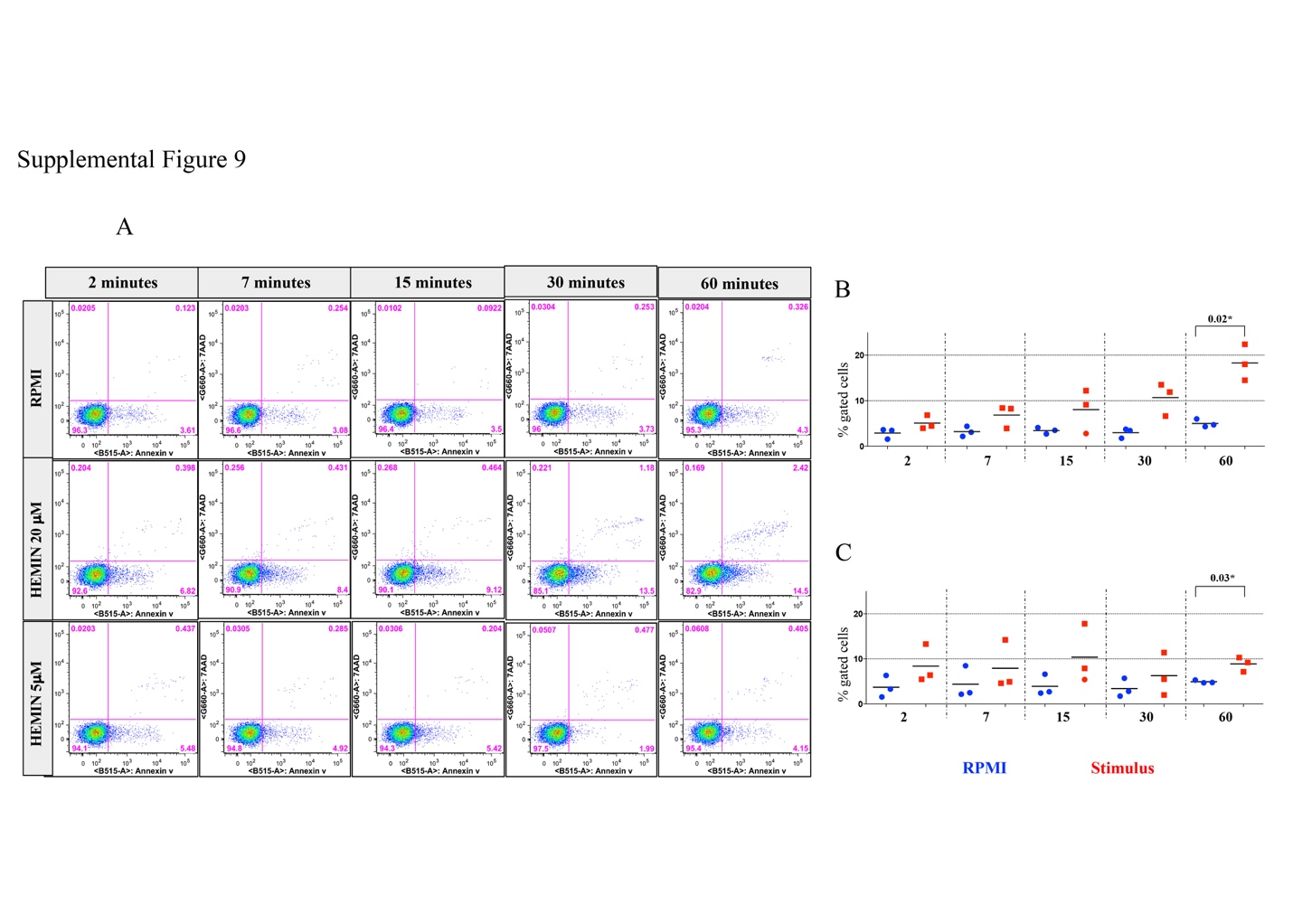
**

**Supplemental Figure 9.** **NETs inducer Hemin causes an increase in Annexin V signal in a time-dependent manner. A**. Representative flow cytometry density plots for Annexin V and 7AAD measurements following treatment Hemin 20 μM and 5 μM for the specified lengths of time. **B**. Changes in the percent Annexin V positive neutrophils following stimulation with Hemin 20 μM. **C**. Changes in the percent Annexin V positive neutrophils after treatment with Hemin 5 μM. Neutrophils from 3 healthy distinct donors were used for experiments. Paired t test was used to compare the response from untreated (blue) and treated (red) neutrophils. *P<0.05.

**
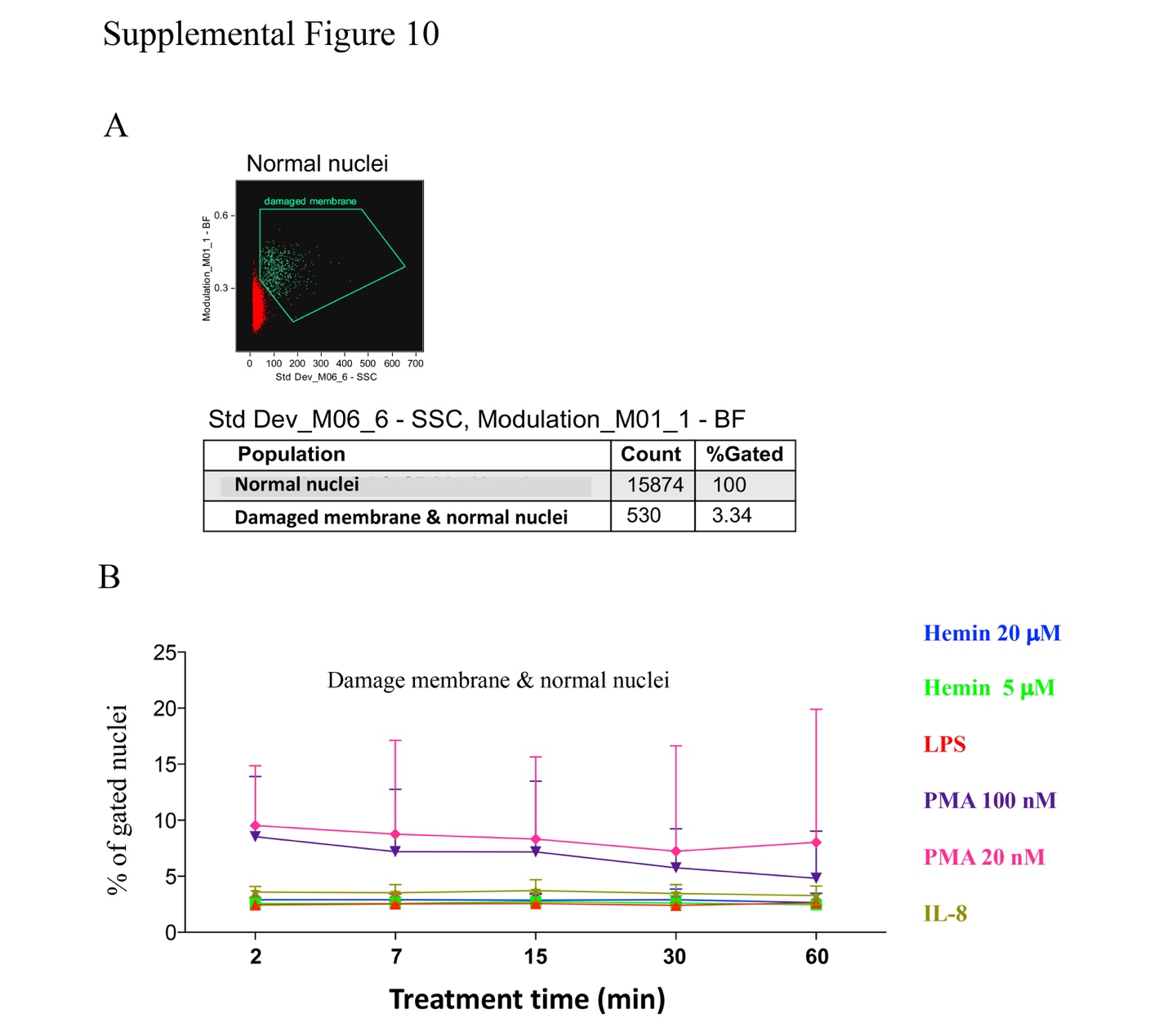
**

**Supplemental Figure 10. NETs stimuli do not cause significant damage to neutrophil cellular membrane. A.** “Standard deviation” and “Modulation” features in IDEAS analysis software identify and quantify cells with damaged plasma cell membrane. **B**. Percent of neutrophils with changes in the cellular membrane remains constant during the treatment at the 5 tested time points.

**Supplemental Table 1. Donors’ contributions to experiments.**

| Donors | Stimulus | | | | | |
| --- | --- | --- | --- | --- | --- | --- |
|  | Hemin 20μM | Hemin  5μM | LPS  1μg/ml | PMA  100 nM | PMA  20 nM | IL-8  50 pg/ml |
| Donor 1 |  | 🗸 | 🗸🗸 | 🗸🗸 | 🗸 |  |
| Donor 2 | 🗸 |  |  | 🗸 |  | 🗸 |
| Donor 3 | 🗸 | 🗸 | 🗸 | 🗸 | 🗸 |  |
| Donor 4 | 🗸 |  | 🗸 |  |  |  |
| Donor 5 |  |  |  | 🗸 |  | 🗸 |
| Donor 6 |  | 🗸 | 🗸 |  |  |  |
| Donor 7 | 🗸 | 🗸 | 🗸 | 🗸🗸 | 🗸 | 🗸 |
